# Multi-omic insights into the cellular response of *Phaeodactylum tricornutum* (Bacillariophyta) strains under grazing pressure

**DOI:** 10.3389/fpls.2023.1308085

**Published:** 2024-01-08

**Authors:** Chenqi Liu, Liang Li, Shuo Yang, Mingye Wang, Hang Zhang, Si Li

**Affiliations:** School of Chemical Engineering, Hebei University of Technology, Tianjin, China

**Keywords:** *Phaeodactylum tricornutum*, grazing stress, transcriptome, proteome, metabolome, phenotype

## Abstract

**Background/Aims:**

*Phaeodactylum tricornutum*, a model organism of diatoms, plays a crucial role in Earth’s primary productivity. Investigating its cellular response to grazing pressure is highly significant for the marine ecological environment. Furthermore, the integration of multi-omics approaches has enhanced the understanding of its response mechanism.

**Methods:**

To assess the molecular and cellular responses of *P.tricornutum* to grazer presence, we conducted transcriptomic, proteomic, and metabolomic analyses, combined with phenotypic data from previous studies. Sequencing data were obtained by Illumina RNA sequencing, TMT Labeled Quantitative Proteomics and Non-targeted Metabolomics, and WGCNA analysis and statistical analysis were performed.

**Results:**

Among the differentially expressed genes, we observed complex expression patterns of the core genes involved in the phenotypic changes of *P.tricornutum* under grazing pressure across different strains and multi-omics datasets. These core genes primarily regulate the levels of various proteins and fatty acids, as well as the cellular response to diverse signals.

**Conclusion:**

Our research reveals the association of multi-omics in four strains responses to grazing effects in *P.tricornutum*. Grazing pressure significantly impacted cell growth, fatty acid composition, stress response, and the core genes involved in phenotype transformation.

## 1 Introduction

Phytoplankton, particularly marine diatoms, are key contributors to Earth’s primary productivity (Shukla et al., 2023), responsible for about one-fifth of the total. The impact of global climate change intensifies the importance of understanding their response to grazing pressure, crucial for marine ecosystem conservation. *Phaeodactylum Tricornutum* (Bowler et al., 2008), a model organism for diatoms, showcases a diverse range of responses to various environmental stressors, including nitrogen and phosphorus deprivation and heavy metal ion exposure (Thamatrakoln, 2021). Moreover, it affects the behavior of its predators (Kim et al., 2022), such as copepods, altering their grazing behavior and consequently, the marine food web. The advent of high-throughput sequencing technologies has allowed for a more in-depth exploration of these responses, extending to many omics levels.

*P.tricornutum*, a pennate diatom with an evolutionary history spanning 90 million years (Sims et al., 2006), displays remarkable plasticity under environmental stressors. In response to nitrogen deprivation, it reorganizes its proteome to facilitate nitrogen removal and minimize lipid degradation, thereby limiting nitrogen use to high-demand pathways (Kang et al., 2023). Phosphorus scarcity triggers cellular changes, increasing polyphosphate production, phosphorus transport, and metalloenzyme production to dissolve organophosphates (Helliwell et al., 2021). The diatom has a defined response to heavy metal ions, where aluminum toxicity targets photosynthesis, inducing increased reactive oxygen species (ROS) and enhancing lipid peroxidation. This effect is mitigated by up-regulated glycolysis and the pentose phosphate pathway, providing the necessary cellular energy and carbon skeletons for growth (Xie et al., 2015).

In the intricate marine food web, the impact of the diatom *P.tricornutum* on its predators, particularly copepods, stands as a significant ecological dynamic. Grazing-induced signals, stemming from mechanical damage or specific compounds, can alter the morphology, physiology, and life history of *P.tricornutum* (Zhang et al., 2021). Toxins produced can impact diatoms’ nutritional quality, thus modulating copepods’ grazing behavior (Park et al., 2023). Furthermore, *P.tricornutum*’s synthesis of aldehydes strategically curtails zooplankton reproduction, effectively regulating predator populations and modulating grazing pressures (Lauritano et al., 2016; Øie et al., 2017; Martino et al., 2019).

High-throughput sequencing technologies have propelled *P.tricornutum* studies to new depths. Detailed transcriptomic and proteomic analyses under low CO_2_ conditions have unearthed profound alterations in metabolic pathways, underscoring changes in carbon acquisition, signaling, and nitrogen metabolism (Clement et al., 2017). Particularly under nitrogen limitation, there is an observable upregulation in nitrogen fixation, central carbon metabolism, and the tricarboxylic acid (TCA) cycle, paralleled by lipid rearrangements favoring triglyceride distribution (Remmers et al., 2018). The advent of high-throughput sequencing technologies has provided an unprecedented opportunity to delve deeper into the intricate biological processes at multiple “omics” levels - namely transcriptomics, proteomics, metabolomics, and non-coding RNAs (Clement et al., 2017; Li and Ismar, 2018; Remmers et al., 2018).

Recent investigations have highlighted substantial variations in growth rate, biovolume, and nutritional composition among individual *P.tricornutum* strains (Li and Ismar, 2018). The sequence replicates of 1055/1 strain corroborates the consistency of these samples. Quantitative simulations and observed shifts in growth rate, biovolume, and nutritional composition have illuminated the effects of copepod grazing pressure on *P.tricornutum*. However, given the genetic diversity and unique response profiles among different strains, a comprehensive understanding of the differential gene expression mechanisms is imperative. Such insights are critical to grasp the species’ adaptive responses to grazing pressure. Therefore, there is a pressing need for extensive, multi-strain, and multi-omics studies to refine our understanding and unravel the gene regulatory networks that drive growth discrepancies and morphological adaptations.

In this study, our objective is to elucidate the genetic underpinnings that govern the diverse strain-specific responses of *P.tricornutum* to grazing pressure. By integrating phenotype- associated gene modules with comprehensive transcriptomic, proteomic, and metabolomic datasets, we aspire to decode the intricate molecular mechanisms driving these responses. This endeavor is not merely an exploration of *P.tricornutum*’s adaptive strategies; it represents a broader initiative to enhance our comprehension of marine ecological interactions. Furthermore, it aims to contribute strategically to the development of informed approaches for mitigating the effects of global environmental changes on these vital ecosystems.

## 2 Materials and methods

### 2.1 Cultivation of *Phaedactylum tricornutum* strains and experimental conditions

Four strains of *P.tricornutum* (CCAP 1052/1A, CCAP 1052/1B, CCAP 1055/3, CCAP 1055/7) were provided by Génomique, Environnementale et Evolutive Section 3 CNRS UMR8197, Institut de Biologie de l’ENS (IBENS). The setting of culture conditions comes from the paper of Li (Li and Ismar, 2018). The culture medium was prepared using sterile-filtered natural seawater (Minisart High-Flow 0.1μm syringe filter; Sartorius Stedim Biotech GmbH, Goettingen, Germany), fortified with macronutrients and micronutrients in accordance with a modified Provasoli medium. *P.tricornutum* cultures were incubated at 18°C under constant illumination of 100 μmol photons·m^-2^·s^-1^, following a 12:12 h light-dark cycle with 2 hours of simulated sunrise and sunset periods.

### 2.2 Experimental design

The design of grazing experiments followed the protocol presented in Li (Li and Ismar, 2018). The grazing pressure was applied using over 2000 *Acartia tonsa* individuals, fed with 1×10^7^ *P.tricornutum* cells daily in 25 L seawater. *Acartia* were maintained in a double-bucket system, with a 100 μm mesh sieve replacing the bottom of the inner bucket (**Supplementary Figure 1**). Daily, seawater in the outer bucket was replaced and *Acartia* faeces and eggs were removed. Grazing treatment replicates were generated by filtering *Acartia*’s culture medium using a 0.1 μm syringe filter and adding it to *P.tricornutum* cultures. Control groups were provides with the same volume of sterile-filtered seawater, maintaining the nutrient ratio of the original medium used for *P.tricornutum* cultivation.

Batch culture experiments were conducted for each *P.tricornutum* strain under two treatments (control and grazing). The 4 strains, under distinct conditions, were grouped into 8 sample sets: pt52_A_c (CCAP 1052/1A strain control group), pt52_A_g (CCAP 1052/1A strain grazing group), pt52_B_c (CCAP 1052/1B strain control group), pt52_B_g (CCAP 1052/1B strain grazing group), pt55_3_c (CCAP 1055/3 strain control group), pt55_3_g (CCAP 1055/3 strain grazing group), pt55_7_c (CCAP 1055/7 strain control group), pt55_7_g (CCAP 1055/7 strain grazing group). Each strain was cultured in 150 ml of medium and subjected to Reference Genome, Transcriptome Sequencing on an Illumina sequencing platform, TMT (Tandem Mass Tag) Labeled Quantitative Proteomics Standard Analysis, and non-targeted metabolomics Bioinformatics Standard Analysis. The grazing treatment groups only received *Acartia* water, ensuring that the responses to the chemical cues from grazing pressure was obtained without within-strain selection due to grazing selectivity.

In a preceding study (Li and Ismar, 2018), three independent experiments were conducted on multiple strains of *P.tricornutum*, and the data demonstrated high repeatability. Rigorous quality control measures were employed to ensure the removal of outliers or any disproportionately impactful data points. Transcriptomic, proteomic, and metabolomic approaches were employed to improve the accuracy of the results. A principal component analysis (PCA) was conducted on the proportion of omics data with K-means clusters among the eight sample groups to examine correlations between different omics within each strain, ensuring mutual validation of data from diverse omics layers (**Supplementary Figure 2**). Employing a multi-level data approach significantly clarifies our analytical results. Despite these constraints, our findings provide valuable preliminary insights and establish a foundational basis for future, more extensive research.

### 2.3 Illumina RNA sequencing

Total RNA was extracted from 4 strains under two conditions (control vs grazing). The quality of the extracted RNA, including its concentration and purity, was confirmed by RNA-specific agarose electrophoresis and Agilent 2100 Bioanalyzer. After the extraction, purification, and library construction processes, Next-Generation Sequencing (NGS) was conducted on the libraries using the Illumina HiSeq sequencing platform, aligning with the Phaeodactylum_tricornutum.ASM15095v2.dna.toplevel.fa reference genome (accessible at https://protists.ensembl.org/Phaeodactylum_tricornutum/Info/Index). The raw sequencing data have been deposited to the NCBI with the dataset identifier PRJNA1008380 (https://www.ncbi.nlm.nih.gov/sra/PRJNA1008380).

Complete transcriptome analysis involved the filtration of raw data and comparison of the filtered high-quality sequence (Clean Data) with the species’ reference genome. Based on the comparison results, the expression level of each gene was determined. After splicing the resulting reads to restore the transcript sequence, they were compared with known mRNA and LncRNA transcripts in order to identify new LncRNA. An analysis of expression differences, target gene enrichment, and cluster analysis was conducted between known LncRNA and newly identified LncRNA. For unmatched sequences, 20bp were intercepted from both ends to obtain Anchor Reads. The Anchor Reads were then re-matched with the genome, and the CircRNA was identified using find_circ based on the comparison results. Then, basic statistical analysis, quantitative and differential expression analysis, functional and pathway enrichment analysis, and miRNA targeting relationship prediction were conducted for CircRNA. The raw miRNA data was filtered for quality, compared with the Rfan and miRBase databases, and various sRNA annotation information was obtained. The characteristics and expression of miRNA were analyzed, and miRNAs with significant differential expression were analyzed using clustering techniques. The predicted target genes were also subjected to enrichment analysis. Differential expression analysis involved standardized mRNA, LncRNA, CircRNA, and miRNA for significant differential expression screening, employing Log2Foldchange treatment and p-value screening. KEGG and GO functional enrichment analysis were employed to ascertain the primary biological functions of the differentially expressed genes.

### 2.4 TMT labeled quantitative proteomics

TMT™ (Tandem Mass Tag™), an *in vitro* labeling technology by Thermo Scientific, was employed to assess relative protein content across our samples. Using labels of 2, 6, 10 or 16 isotopes, peptides’ amino groups were specifically labeled, followed by tandem mass spectrometry to analyze protein content variation in 2, 6, 10, or 16 groups simultaneously.

Our eight samples (four strains) from the two treatments (control and grazing) underwent protein extraction through the TCA acetone precipitation method, and SDT (4%(w/v) SDS, 100mM Tris/HCl pH7.6, 0.1M DTT) cleavage method (Wisniewski et al., 2009). Then the protein was quantified by BCA method. 200μg of protein from each sample was used for tryptic enzymatic hydrolysis via the Filter aided proteome preparation (FASP) method (Wisniewski et al., 2009), followed by enzymatic enzymatic peptide desalination using C_18_ Cartridge. The desalted peptide was then lyophilized and dissolved with a 40μL Dissolution buffer (OD280). Subsequent to this, 100μg of the peptide segment from each sample was labeled using the TMT labeling kit (Thermo Fisher), and the proteins were identified and quantitatively analyzed after SCX chromatography and LC-MS/MS data collection. The mass spectrometry proteomics data have been deposited to the ProteomeXchange Consortium (http://proteomecentral.proteomexchange.org) via the iProX partner repository (Ma et al., 2019; Chen et al., 2021) with the dataset identifier PXD044954.

### 2.5 Non-targeted metabolomics

HILIC UHPLC-Q-EXACTIVE MS technology coupled with a data-dependent acquisition method was adopted for full-spectrum analysis of the samples. Compound Discoverer 3.0 (Thermo Fisher Scientific) facilitated peak extraction and metabolite identification (Benton et al., 2015). The chromatographic separation of the extracted metabolites from the eight samples was performed using an ACQUITY UPLC BEH C18 column (100 mm*2.1 mm, 1.7μm, Waters, USA) with a column temperature set at 40°C and a flow rate of 0.3 ml/min. Both positive and negative ion modes of electrospray ionization (ESI) were utilized for detection. The samples post- UHPLC separation were analyzed with a Q-Exactive four-pole and a Thermo Fisher Scientific mass spectrometer. The resulting raw mass spectrometry data were processed by Compound Discoverer 3.0 (Thermo Fisher Scientific) software for peak extraction, peak alignment, peak correction, and standardization, resulting in a 3D data matrix composed of the sample name, spectral peak information (including retention time and molecular weight), and peak area. The metabolite structure was identified by precise mass number matching (<25 ppm) and secondary spectrogram matching method to search the laboratory database and various other databases including Bio cyc, HMDB, Metlin, HFMDB, Lipidmaps. The metabolomics data have been deposited to the Metabolights (Haug et al., 2020) with the dataset identifier MTBLS8485 (www.ebi.ac.uk/metabolights/MTBLS8485).

### 2.6 Weighted gene co-expression network analysis

Co-presentation network construction employed the WGCNA (Version 1.71) (Langfelder and Horvath, 2008). Hierarchical clustering of the sample data was first conducted to calculate the test of delocalization value. During co-expression network construction, we screened soft threshold values. Hierarchical clustering and dynamic tree cutting algorithms (Langfelder et al., 2008) were used to group genes with similar expression patterns and categorize them into different gene co-expression modules. We used the Pearson correlation coefficient to assess the relationship between the characteristic gene (Pei et al., 2017) of the gene co-expression module and the phenotype. Hierarchical clustering of the sample data was carried out and the Pearson correlation coefficient was calculated to link the sample to the phenotype.

In WGCNA, the core genes within each co-expression module can be evaluated by Gene Significance and Module Membership index (Pei et al., 2017). In this study, the genes with |KME|≥0.8 and |GS|≥0.8 in each module were identified as the core genes in the module. We then used the Gene Significance between genes and phenotypes within each module to map the Connectivity of genes within the module to verify the correlation between modules and phenotypes. The essential code for WGCNA analysis is available in the appendix for reference.

### 2.7 Softwares

Statistical analysis were performed using RStudio (R Core Team, 2022).

## 3 Results

### 3.1 Transcriptome profiling of *P.tricornutum*

The genomes of 8 samples of *P.tricornutum* were sequenced using the Illumina HiSeq sequencing platform. Following the removal of adapter and the filteration of low-quality sequences, the resultant clean reads counts were as follows in **Table 1**. For accurate genome alignment, clean reads were mapped to the reference genome ASM15095v2 using the BWT algorithm implemented in the HISAT2 software. The mapping efficiencies for each sample are detailed in **Supplementary Table 2**.

**Table 1:** Basic information about the sequencing data after data filtering.

Sample	Reads No.	Clean Read No.	Bases (bp)	Clean Data (bp)	Clean Reads %	Clean Data %
pt52_A_c	107600456	98856954	16140068400	14828543100	91.87	91.87
pt52_A_g	118828944	109112418	17824341600	16366862700	91.82	91.82
pt52_B_c	109030966	99613970	16354644900	14942095500	91.36	91.36
pt52_B_g	110369976	100799184	16555496400	15119877600	91.32	91.32
pt55_3_c	106200518	96171702	15930077700	14425755300	90.55	90.55
pt55_3_g	104258976	94442576	15638846400	14166386400	90.58	90.58
pt55_7_c	121164860	107697388	18174729000	16154608200	88.88	88.88
pt55_7_g	114128898	103032006	17119334700	15454800900	90.27	90.27

Reads No.: Total Reads; Clean Read No.: High quality sequence read number; Bases (bp): Total number of bases; Clean Data (bp): High quality sequence base number; Clean Reads %: High quality sequence reads accounted for the percentage of sequencing reads; Clean Data %: High quality sequence bases accounted for the percentage of sequencing bases.

**Figure 1:**
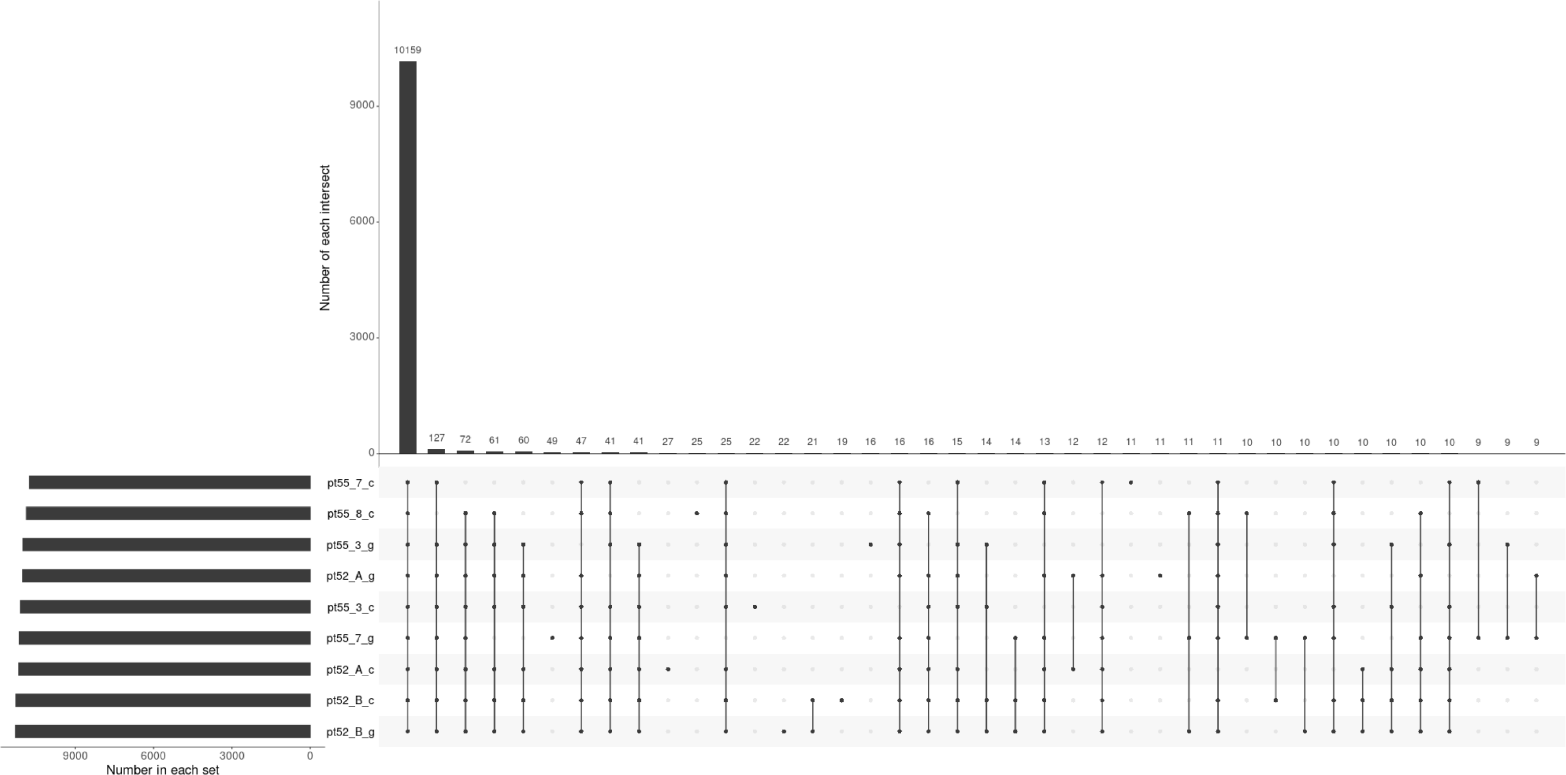
Upset plot representing gene identification across samples. Each set’s cardinality represents the total count of genes identified within that particular sample. Intersection cardinalities denote the number of genes detected in multiple samples. The horizontal line connecting all points signifies the universally identified genes across all samples, while the remaining lines, connecting single or multiple points, represent genes exclusive to their respective samples.

**Figure 2:**
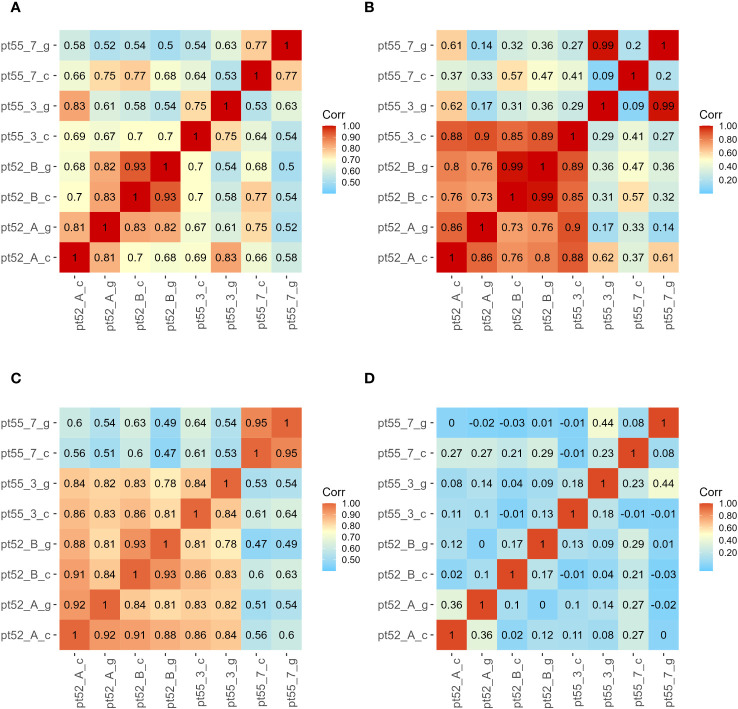
Sample correlation Analysis. Sample identifiers are presented on the left and bottom. The color gradient within each square quantifies the correlation strength between corresponding sample pairs. Subfigure (A) corresponds to mRNA. Subfigure (B) denotes LncRNA. Subfigure (C) represents CircRNA. Subfigure (D) highlights miRNA.

**Figure 3:**
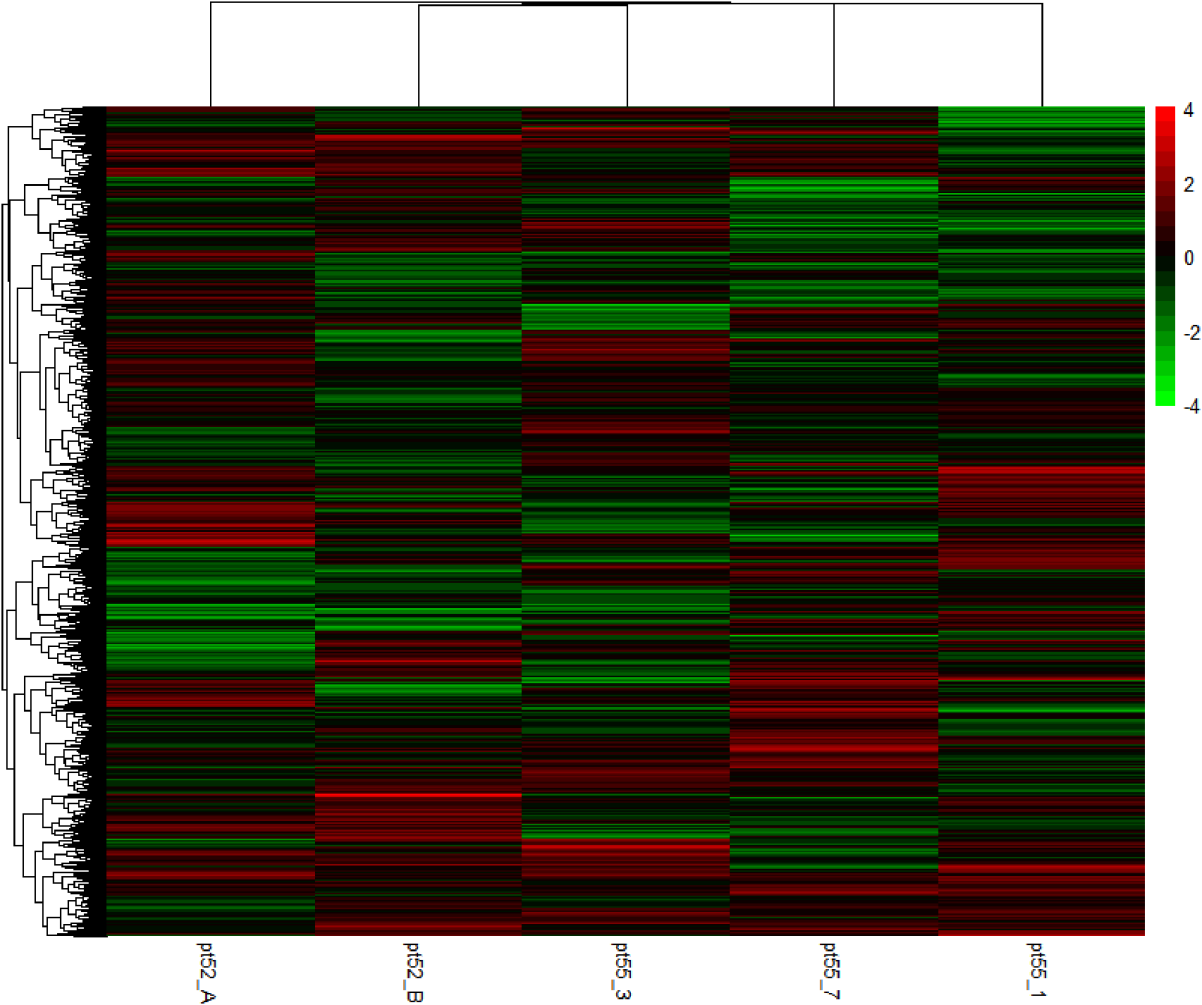
Cluster analysis of heat map of DEGs. Green bars represent the down-regulated genes. Red bars represent the up-regulated genes. The color depth represents the log2 ratio.

The mRNA expression of *P.tricornutum* was normalized using the Fragments Per Kilo base of transcript per Million mapped reads (FPKM) metric across various genes and samples. This study identified12392 mRNA genes. Gene prevalence in each sample was enumerated based on mRNA expression profiles, with the statistical interplay of unique and common genes across all samples illustrated in **Figure 1**. The inter-sample correlation, based on FPKM values, revealed varying degrees of differential expression amongst the four strains under grazing stress (**Figure 2**). Notably, the correlation coefficient between the control and grazing groups for all four strains exceeded 0.75, with strain pt52_B exhibiting the highest correlation (0.93), indicating relatively lesser differential expression under grazing pressure. Differential gene expression analysis, conducted using DESeq, adhered to criteria of |log2FoldChange| >1 and P-value < 0.05. This approach identified 1235 mRNA genes as significantly differentially expressed. Comparative analysis between control and grazing pressure groups uncovered distinct expression patterns for the four strains under grazing stress, with additional expression data from the CCAP1055_1 strain represented in **Figure 3**.

For LncRNA gene expression, FPKM normalization was similarly differences applied. Our analysis identified a total of 505 LncRNAs, with correlation analysis indicating varied degrees of differential expression among the strains under grazing pressure. Notably, between pt52_B_c and pt52_B_g were statistically insignificant, while pronounced differences were observed in the pt55_3_c vs pt55_3_g and pt55_7_c vs pt55_7_g pairings, as evidenced in **Figure 2**. Differential analysis of LncRNA expression set the criteria for differentially expressed genes at |log2FoldChange| >1 and P-value < 0.05. After this filtration, 65 LncRNA transcriptional genes showed significant differential expression, and cis-acting target gene prediction was performed for these genes, with corresponding target mRNAs identified (the relationships are delineated in the **Supplementary Table 3**).

For CircRNA expression, normalization employed the Transcripts per Million of a gene (TPM) metric, identifying a total of 536 CircRNAs. Inter-sample expression correlations, based on TPM, indicated varied differential expressions across strains under grazing pressure, with no significant variations among control groups (**Figure 2**). Despite using consistent differential expression criteria of |log2FoldChange| > 1and P-value < 0.05, no CircRNA showcased significant differential expression upon further analysis.

miRNA expression was normalized using the Counts per Million (CPM) metric, identifying 297 known miRNAs. Correlation analyses of sample expressions, based on CPM, showed notably low relationships among the four strains under grazing stress (**Figure 2**).

### 3.2 Proteomic profiling of *P.tricornutum*

In our comprehensive proteomic analysis of eight *P.tricornutum* samples, 4984 proteins and 22985 peptides were identified. Proteins exhibiting differential expression, defined by an expression alternation exceeding 1.5-fold (upregulated beyond 1.5 times or downregulated below 0.67-fold), are detailed in **Table 2**.

**Table 2:** Protein quantification and differences.

Comparisons	Up	Down	All
1052_1A_c VS 1052_1A_g	134	128	262
1052_1B_c VS 1052_1B_g	116	202	318
1055_3_c VS 1055_3_g	227	389	616
1055_7_c VS 1055_7_g	434	326	760

To glean deeper biological insights, significant differentially expressed proteins were subjected to KEGG pathway enrichment analysis, the details of which are presented in **Supplementary Table 5**. This proteomic exploration was further enriched by integrating mRNA, LncRNA, and metabolomic data for an encompassing metabolic pathway enrichment and analysis.

### 3.3 Metabolomic profiling of *P.tricornutum*

Metabolomic analysis of eight *P.tricornutum* samples, using Compound Discoverer 3.0 software, yielded 5967 positive ion peaks and 5814 negative ion peaks. Noteworthy Total Ion Chromatogram (TIC) patterns emerged from the Mass Spectrometry (MS) evaluation. The significant differential metabolites were clustered and enriched by KEGG pathway (**Supplementary Table 6**). This analysis was further integrated with mRNA, LncRNA, and proteomic data to perform comprehensive metabolic pathway enrichment and analysis.

### 3.4 Pathway enrichment and analysis

**Figure 4:**
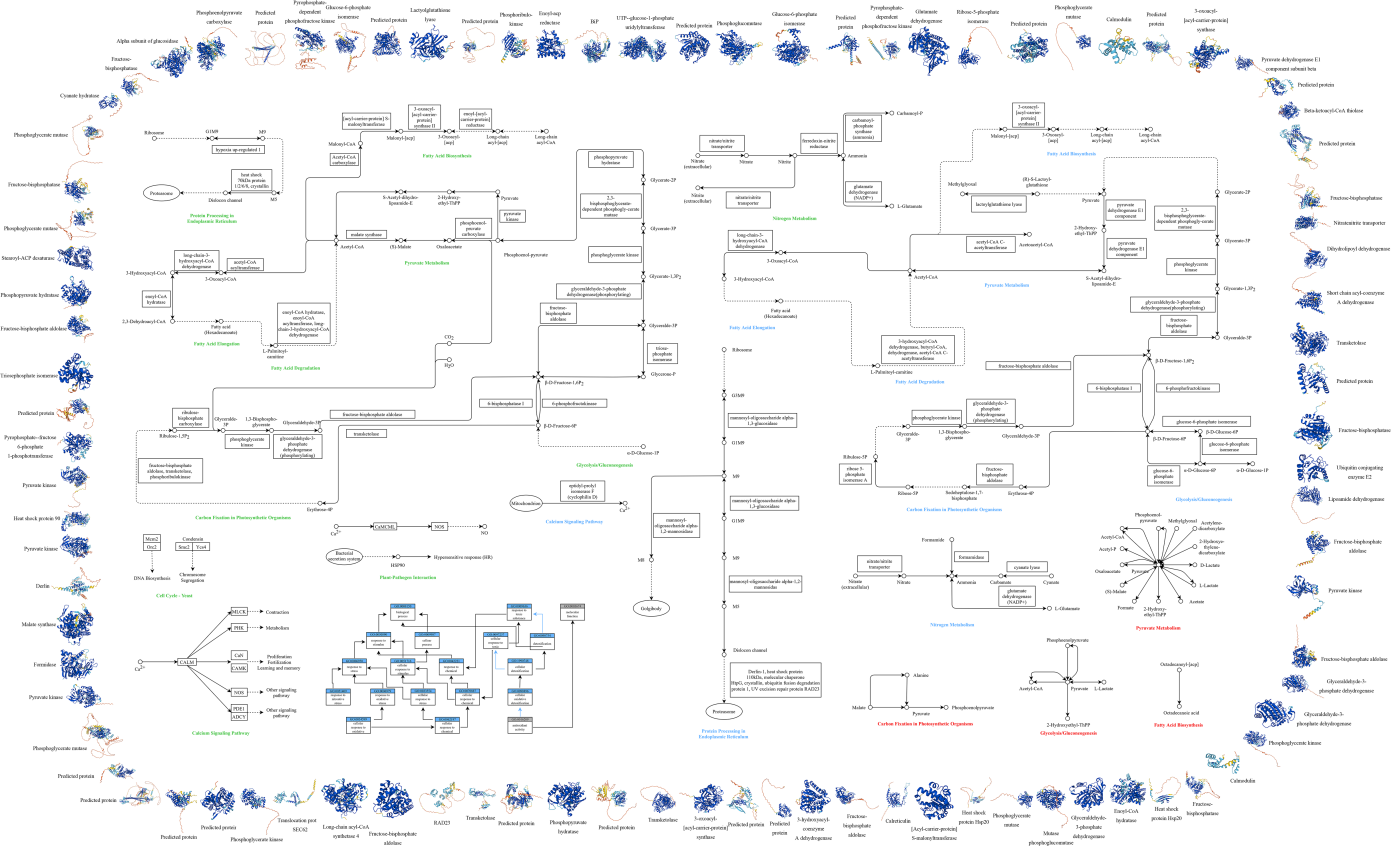
Multi-omics Cell Function Map of P.tricornutum. This figure illustrates the integrated analysis of transcriptomics, proteomics, and metabolomics, along with GO and KEGG pathway analysis, highlighting the significant variations under grazing pressure. Outer Ring: The periphery of the diagram represents the protein structure involved, color-coded by Model Confidence according to the AlphaFold’s per-residue confidence score (pLDDT): Blue: pLDDT > 90, Cyan: 70 ≤ pLDDT ≤ 90, Yellow: 50 ≤ pLDDT ≤ 70,Orange: pLDDT < 50. Regions with low pLDDT may signify isolated or unstructured segments. Middle Section: Encompasses GO and KEGG pathway analysis for P.tricornutum under grazing pressure, integrating transcriptomics, proteomics, and metabolomics: GO Analysis (Lower Left Corner): Blue rectangles indicate Process IDs and names; gray rectangles indicate Function IDs and names; black arrows represent “is” relationships; blue arrows denote “part of” relationships. KEGG Metabolic Pathway Analysis (Remaining Middle Section): Circles: Various compounds, DNA, or other molecules; Solid Arrows: Direct interactions or relationships; Dashed Arrows: Indirect connections or unidentified reactions; Double Arrows: Reversible conversions; Rectangles: Required genes or enzymes; Ellipses: Specific organelles; Font Colors: Represent the metabolic pathways obtained through different analyses - green for transcriptomic, blue for proteomic, and red for metabolomic.

Significantly differentially expressed genes within *P.tricornutum* mRNA under grazing pressure were aligned with both Gene Ontology (GO) and KEGG metabolic pathways (**Figure 4**). This revealed prominent involvement in pathways such as Glycolysis and Gluconeogenesis, Fatty Acid Biosynthesis, the Fatty Acid Elongation, Fatty Acid Degradation and Pyruvate Metabolism, Protein Processing in Endoplasmic Reticulum, Carbon Fixation in Photosynthetic Organisms, Nitrogen Metabolism. Furthermore, pathways linked with stress responses, including Calcium Signaling, Oxidative Stress, Nitrosative Stress, and Antioxidant Activity, were also noteworthy. The specific data of KEGG Pathway Enrichment in Transcriptomic (**Supplementary Table 7**), Proteomic (**Supplementary Table 5**) and Metabolomic (**Supplementary Table 6**) are in the Appendix.

**Figure 5:**
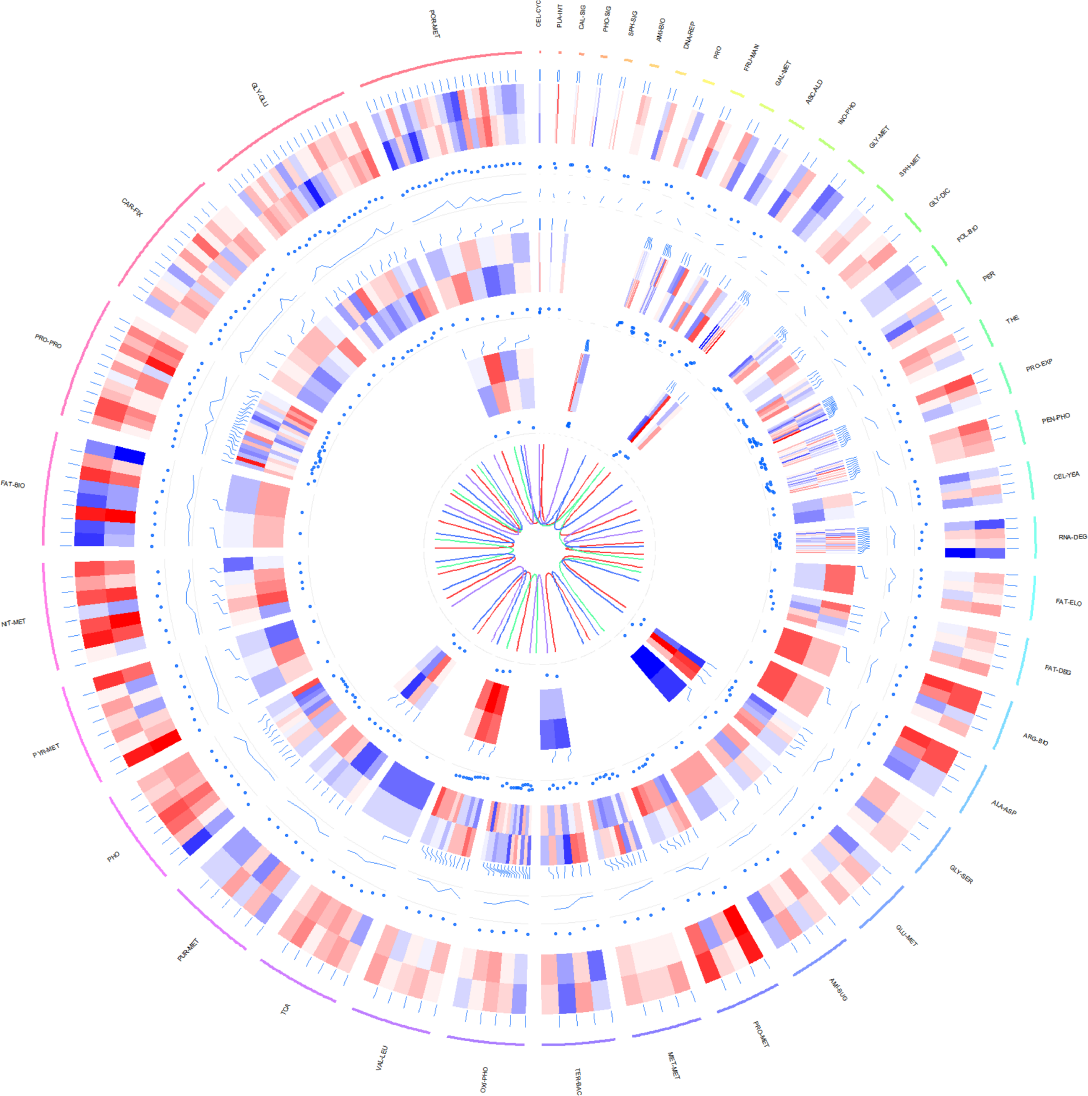
Circos plot illustrating differential expression across transcriptomic, proteomic, and metabolomic data in P.tricornutum’s KEGG pathway under grazing stress. Transcriptomic Data: Represents the log10-transformed gene transcription levels for each KO. Proteomic Data: Demonstrates the log2-transformed expression levels of proteins, comparing the grazing pressure group with the control group. Metabolomic Data: Features the log10-transformed expression profiles of metabolites, contrasting the grazing pressure and control groups. From the outside in: The first circle is the metabolic path corresponding to each gene and its text annotation, the bar color is meaningless; The second circle is a heat map of the data expression at the transcriptomic level in the metabolic pathway (red to blue with red representing up-regulation and blue representing down-regulation); The third circle is the dot plot of differential expression values of each gene corresponding to the grazing pressure group and the control group in transcriptomic level. The fourth circle is the line chart of the P-Value of each gene in the transcriptomic level; The fifth circle is a heat map of data expression at the proteomic level of metabolic pathways (color meaning is the same as transcriptomic data); The sixth circle is the dot plot of the differential expression values of each gene corresponding to the grazing pressure group and the control group in the proteomic level. The seventh circle is a heat map of data expression at the metabolomic level in the metabolic pathway (color meaning is the same as the transcriptomic data); The eighth circle is the dot plot of the differential expression values of each gene corresponding to the grazing pressure group and the control group in the data of the metabolomic level. The ninth circle is the relationship diagram of the four strains distinguished by color, where brown represents pt52_A, seagreen represents pt52_B, royalblue represents pt55_3, and mediumpurple represents pt55_7. The outermost metabolic path annotation is: CEL-CYC (Cell cycle); PLA-INT (Plant-pathogen interaction); CAL-SIG (Calcium signaling pathway); PHO-SIG (Phosphatidylinositol signaling system); SPH-SIG (Sphingolipid signaling pathway); AMI-BIO (Aminoacyl-tRNA biosynthesis); DNA-REP (DNA replication); PRO (Proteasome); FRU-MAN (Fructose and mannose metabolism); GAL-MET (Galactose metabolism); ASC-ALD (Ascorbate and aldarate metabolism); INO-PHO (Inositol phosphate metabolism); GLY-MET (Glycerophospholipid metabolism); SPH-MET (Sphingolipid metabolism); GLY-DIC (Glyoxylate and dicarboxylate metabolism); FOL-BIO (Folate biosynthesis); PER (Peroxisome); THE (Thermogenesis); PRO-EXP (Protein export); PEN-PHO (Pentose phosphate pathway); RNA-DEG (RNA degradation); FAT-ELO (Fatty acid elongation); FAT-DEG (Fatty acid degradation); ARG-BIO (Arginine biosynthesis); ALA-ASP (Alanine, aspartate and glutamate metabolism); GLY-SER (Glycine, serine and threonine metabolism); GLU-MET (Glutathione metabolism); AMI-SUG (Amino sugar and nucleotide sugar metabolism); PRO-MET (Propanoate metabolism); MET-MET (Methane metabolism); TER-BAC (Terpenoid backbone biosynthesis); OXI-PHO (Oxidative phosphorylation); VAL-LEU (Valine, leucine and isoleucine degradation); TCA (Citrate cycle (TCA cycle)); PUR-MET (Purine metabolism); PHO (Photosynthesis); PYR-MET (Pyruvate metabolism); NIT-MET (Nitrogen metabolism); FAT-BIO (Fatty acid biosynthesis); PRO-PRO (Protein processing in endoplasmic reticulum); CAR-FIX (Carbon fixation in photosynthetic organisms); GLY-GLU (Glycolysis/Gluconeogenesis); POR-MET (Porphyrin metabolism).

Concurrently, genes markedly differentially expressed across the transcriptome, proteome and metabolome of *P.tricornutum* under grazing conditions were compared against the KEGG metabolic pathway (**Figure 5**).

### 3.5 Weighted gene co-expression network analysis

Utilizing the expression data of 12802 selected genes across eight samples, a weighted gene co-expression network was constructed.

**Figure 6:**
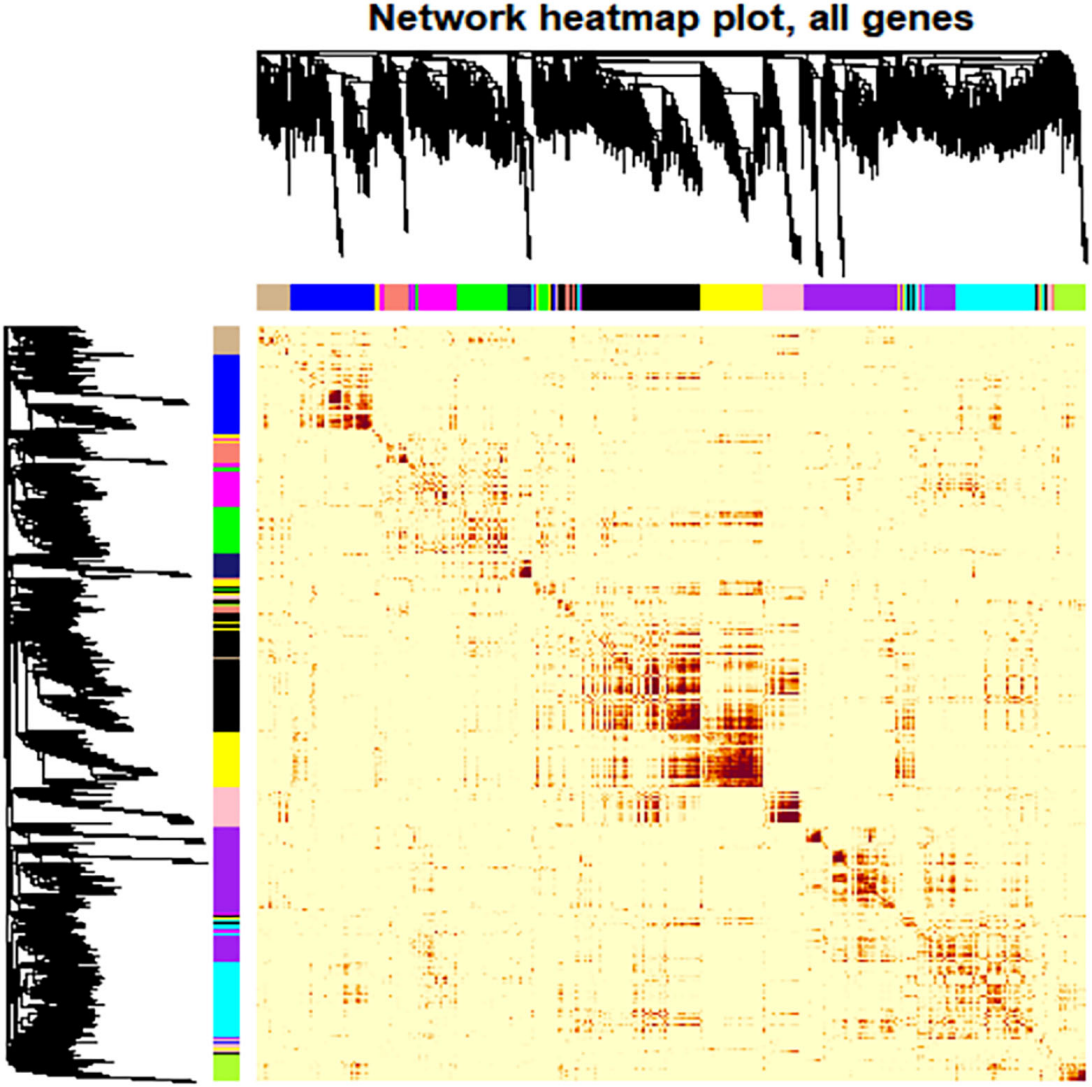
Heat map depiction of the topological overlapping matrix (TOM). This visualization represents the gene values within the TOM, providing insights into gene interactions within and across modules. Hierarchical Clustering and Modules: The upper and left sides of the graph depict a hierarchical clustering tree and the corresponding core presentation modules identified by a dynamic tree cutting algorithm. TOM Values: The color gradient within the figure illustrates the TOM values, while progressively darker shades indicate higher value. Interpretation: The most profound darkness near the diagonal line signifies the strongest gene interaction within individual modules. In contrast, The darker regions further from the diagonal reveal interactions between corresponding modules.

Network Construction: A cluster analysis was performed on the eight samples to identify outlying values. A soft threshold was determined to achieve a scale-free topological fit coefficient (R²) of 0.8 (**Supplementary Figure 8**). Hierarchical clustering was employed to segregate genes into modules based on their correlations, followed by merging modules exhibiting similar expression patterns. Ultimately, 13 distinct modules were identified, ranging in size from 11 to 2066 genes, each represented by different colors (**Supplementary Table 8**, **Figure 6**).

**Figure 7:**
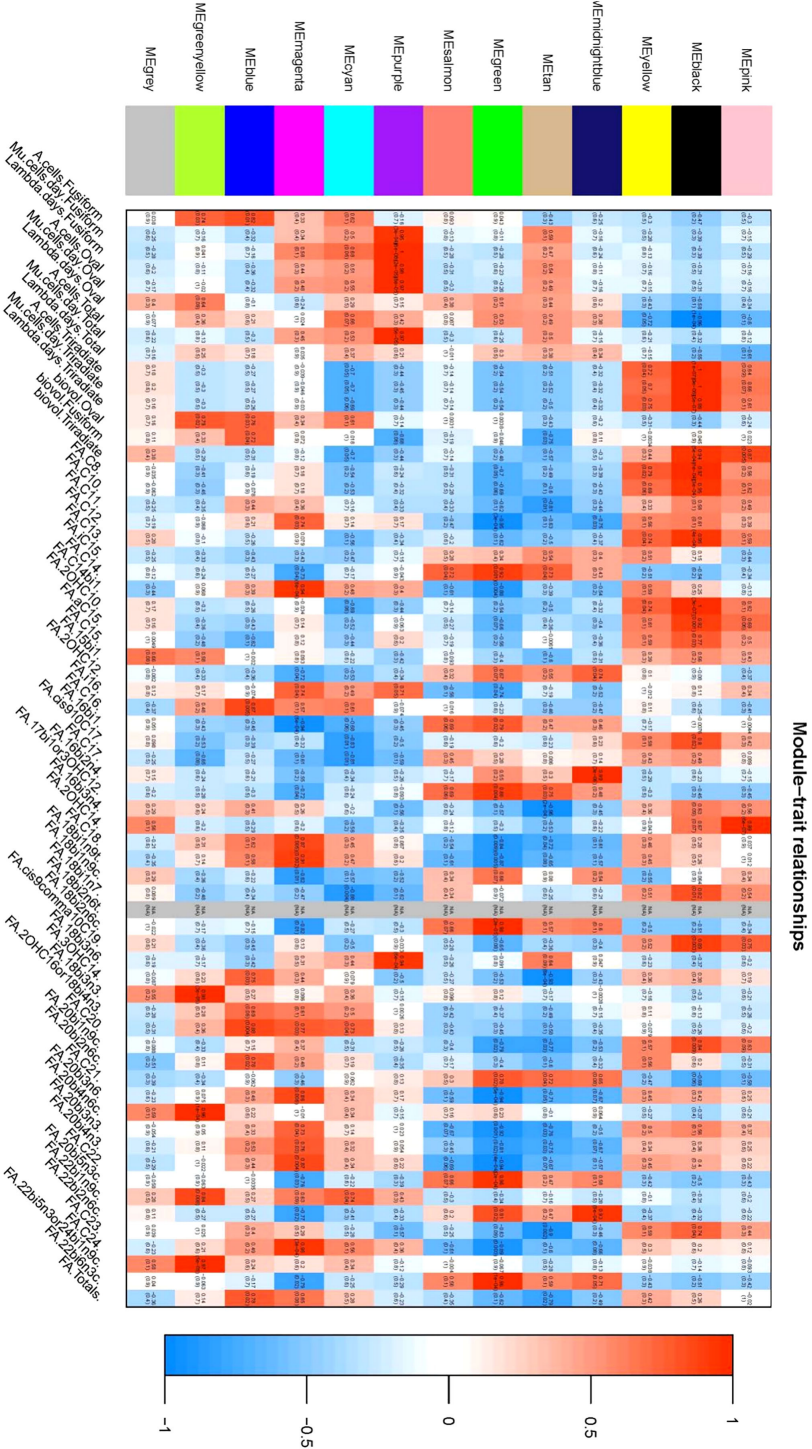
Heat map of correlation illustrating the correlation between coexpression modules and phenotypes: This figure delineates the relationship between individual coexpression modules and specific phenotypes. The X-axis represents individual co-expression modules, depicted in the left color block, while the Y-axis represents all phenotypes, shown on the lower side of the figure. within the graph, color coding is used to represent the correlation values. Red indicates a positive correlation, blue denotes a negative correlation, and white signifies no correlation. Each cell within the figure contains two values; The upper value is the Pearson correlation coefficient, and the lower value represents the P-value. the figure elucidates that the two co-expression modules most positively correlated with each phenotype are identified as the black module and the purple module.

**Figure 8:**
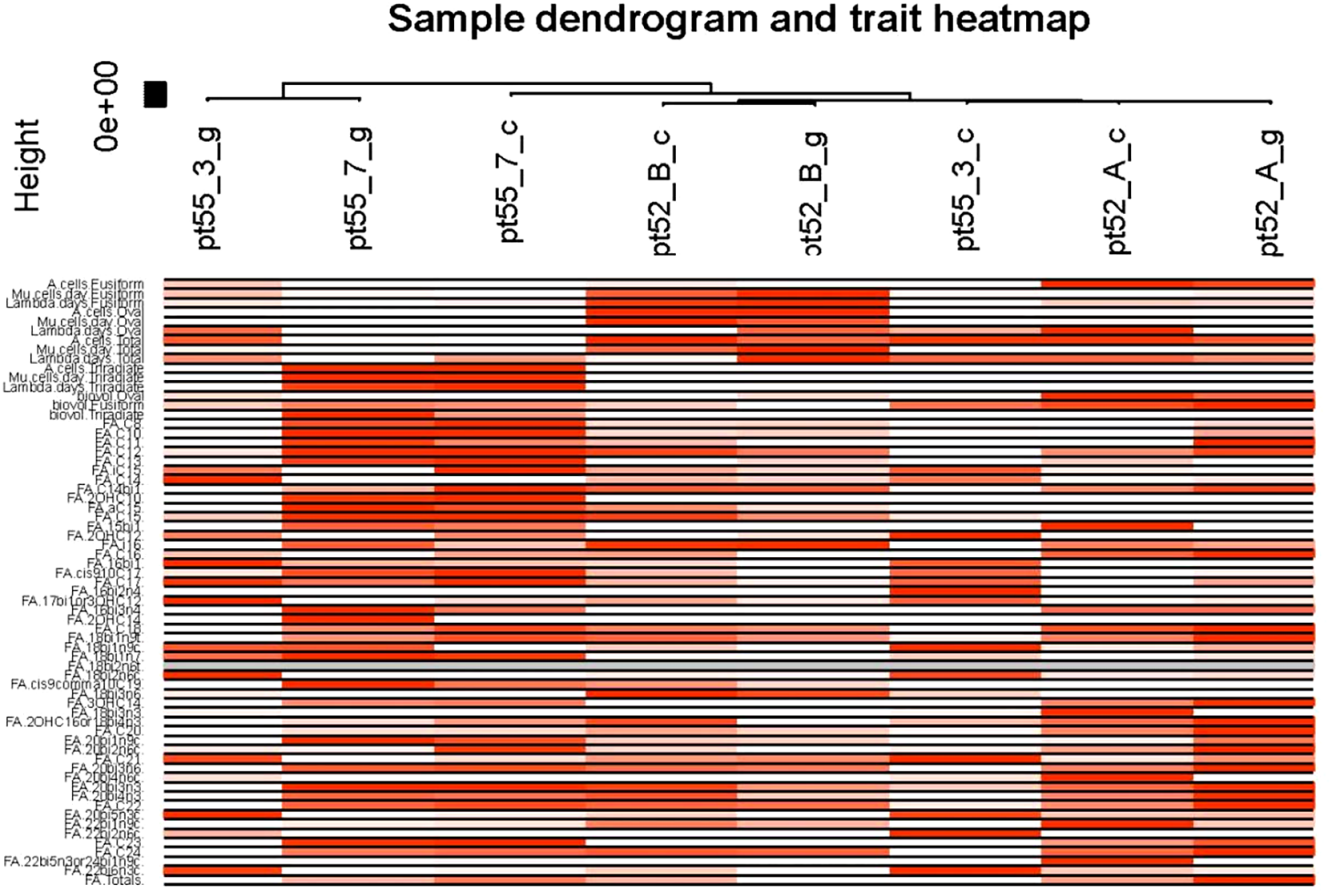
Sample and phenotypic correlation heat map. The X-axis designates the samples while the Y-axis illustrates various phenotypes. Progressive shades of red reflect the strength of the correlation between samples and phenotypes. White signifies the absence of correlation, while gray denotes missing data points.

Phenotypic Association and Interaction Analysis: Phenotypic data were associated with gene co-expression modules following outlier detection. The characteristic genes within each module were considered as representatives for module-specific expression profiles. Correlations between module characteristic genes and various phenotypes were computed, providing insights into the relationships within gene co-expression modules (**Figures 7**, **8**).

**Figure 9:**
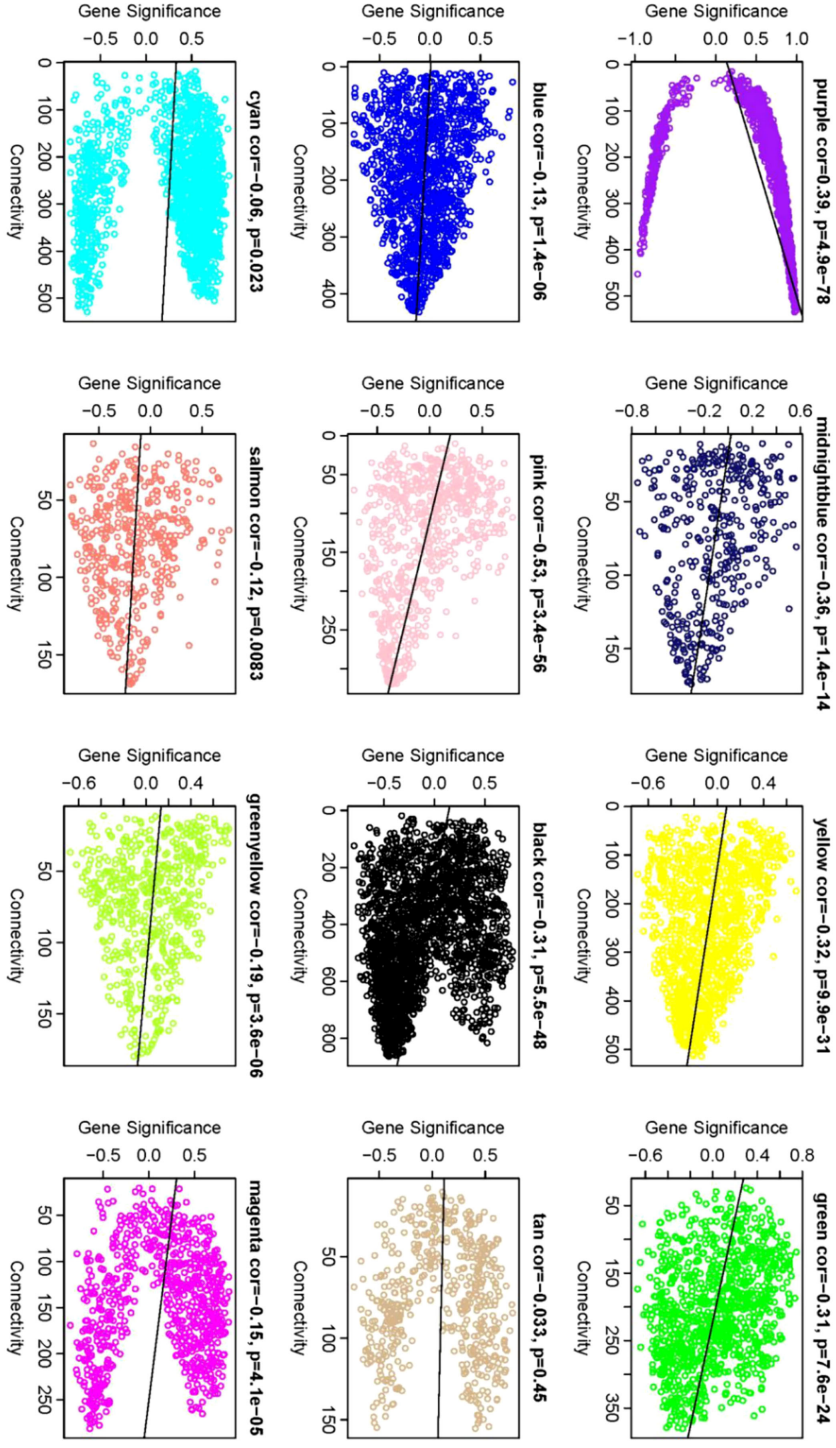
Gene significance vs. connectivity scatter plot. The scatter plot illustrates the relationship between a gene’s significance relative to phenotype and its connectivity within modules. Each data point corresponds to an individual gene. Notably, core genes display heightened |GS| values and increased connectivity. Therefore, the genes located in the upper and lower right corners are classified as core genes. This visual allows for the discernment of modules most closely correlated with phenotype, providing complementary information to that depicted in **Figure 7**.

Identification of Core Genes: Within the network, Gene Significance (|GS|) and Module Membership (|KME|) indices of ≥0.8 were used to ascertain core genes within modules. A comprehensive analysis was conducted to identify the module with the highest phenotype correlation. Correlations between phenotypes and co-expression modules were further validated by mapping gene connectivity within each module (**Figure 9**).

KEGG Pathway Annotation: The R KEGGREST Package (Version 1.36.0) was employed for annotation, screening with a significance level of p < 0.05 (**Supplementary Table 9**). A total of 29 phenotypic core genes were annotated across 38 pathways, with varying correlations to distinct phenotypic modules.

**Figure 10:**
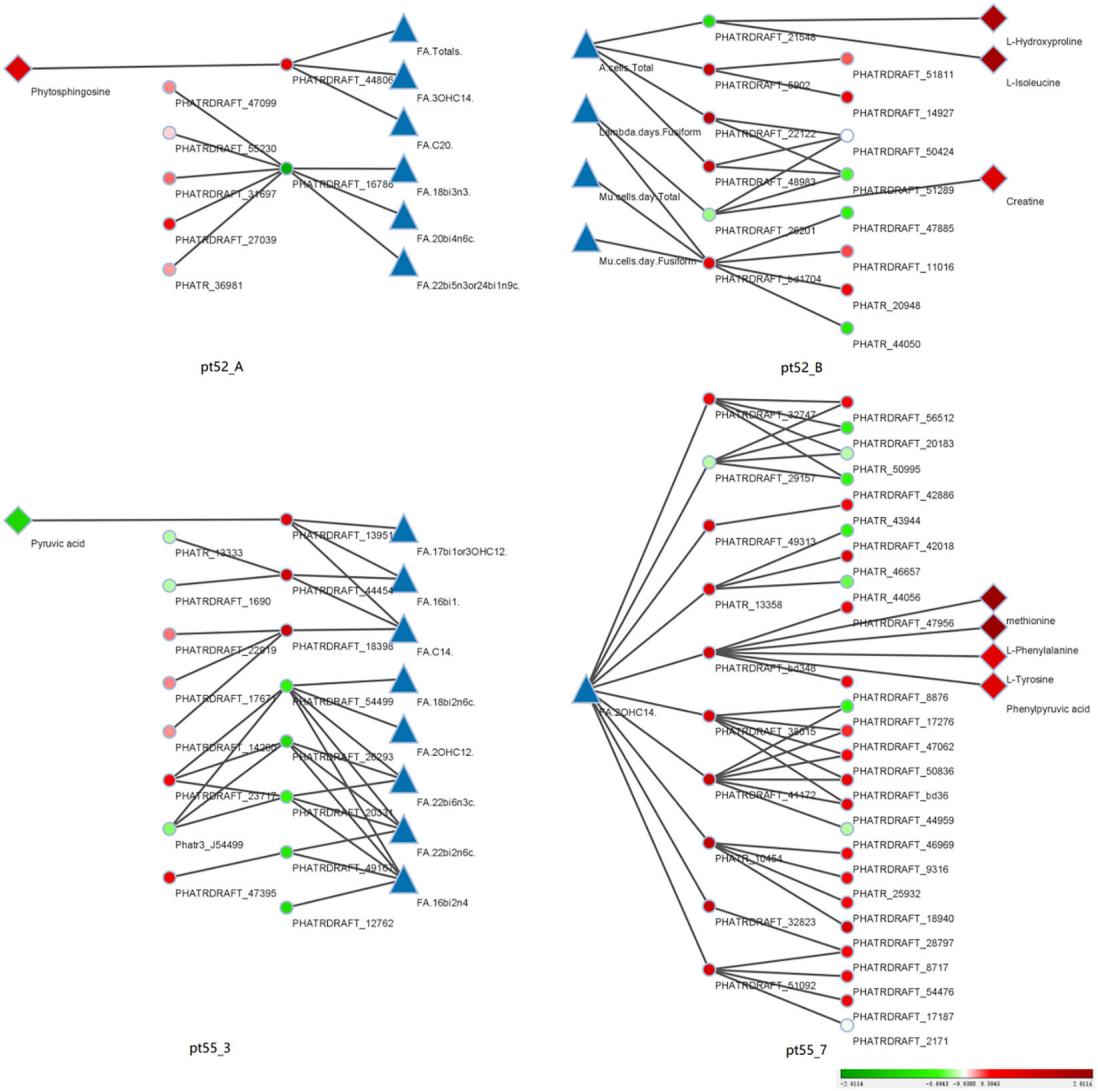
Coexpression relationships across phenotypic data: This figure portrays the coexpression interactions among core genes, transcriptional genes, proteins, and metabolites across various phenotypes, as discerned through WGCNA. Triangles represent distinct phenotypic data. Dots represent individual transcriptional gene IDs or protein gene IDs. Diamond symbolize specific metabolite names. Lines joining the icons denote co-expression relationships. A gradient from red to green depicts the degree of differential expression of transcriptional genes, protein genes or metabolites. Intense shades of red indicate significant up-regulation, while deeper greens indicate pronounced down-regulation. White suggests negligible differential expression. Blue is not representative of any specific data. The right periphery of each icon is labeled with its respective name. Additionally, each of the four segments is labeled with a specific strain name. The sequence from left to right consists of metabolites, protein genes, transcription genes, phenotypic data, and is mirrored for the right half of the figure.

Integration of Multi-Omics Data: The core genes were compared and associated with the transcriptome, proteome, and metabolome. The resulting multi-omics co-expression network was visualized via Cytoscape (Version 3.9.1), elucidated in **Figure 10**.

## 4 Discussion

### 4.1 Transcriptomic insights and omics convergence in *P.tricornutum* Strains under grazing pressure

In our exploration of the transcriptome across four *P.tricornutum* strainsunder grazing pressure, a discernible convergence emerged: genes consistently exhibited up-regulation or down-regulation, albeit with varying intensities. While there are shared defensive responses among strains when exposed to herbivorous stressors, distinct transcriptomic regulatory mechanisms also exist across different strains. Notably, the pt55_1 strain from the prior studies (Li and Ismar, 2018) exhibited transcriptomic patterns similar to those of the pt55_3 strain in our analysis.

The advancement in omics methodologies, notably RNA, protein, and metabolic level analyses (Blum et al., 2022; Cheng et al., 2023), has allowed for a more integrated and comprehensive examination of complex networks, from genes to phenotypes. The omic patterns observed in *P.tricornutum* strains underpin a nuanced understanding of organismal responses to external pressures.

Notably:

Cellular Processes: A uniform down-regulation of genes was observed at the transcriptomic level, while the proteome exhibited an equitable distribution of up- and down-regulated genes, demonstrating overall subdued differential expression.

Organic Systems and Environmental Information Processing: Consistent up-regulation of genes was noted at both transcriptomic and proteomic levels, with a more tempered up-regulation in the proteome compared to the transcriptome.

Genetic Information Processing: A stark contrast between the transcriptome and proteome emerged, with most transcriptomic alterations showing down-regulation, whereas the proteome predominantly exhibited up-regulation. The metabolome added another dimension, displaying varied degrees of up-regulation in significantly altered genes.

Metabolism Pathways: Under grazing stress, *P.tricornutum* employed diverse gene regulatory strategies across different metabolic pathways as a defense mechanism. Noteworthy trends include: down-regulation across both transcriptomic and proteomic dimensions in the Cell Cycle pathway, with a more subdued effect at the protein level; down-regulation in pathways related to Plant-pathogen interaction and Calcium signaling, in response to grazing pressures; contrasting trends in Aminoacyl-tRNA biosynthesis with down-regulation at the transcriptome but pronounced up-regulation at the metabolome; varied gene expression in Terpenoid backbone biosynthesis across transcriptome and proteome; yet a unified up-regulation in the metabolome. In sum, the contrasting gene expressions across omics, especially in pathways like Valine, leucine, and isoleucine degradation, reaffirm the intricate and multifaceted nature of gene regulation in *P.tricornutum* under grazing pressure.

### 4.2 Mechanisms of cellular response under grazing pressure in *P.tricornutum* Strains

#### 4.2.1 Cellular processes

Cellular Processes primarily reflect alterations in the abundance, size, and cell cycle of *P.tricornutum* cells in response to grazing pressure. A consistent up-regulation in genes associated with protein synthesis was observed across different strains at both the transcriptomic and proteomic levels. In particular, triradiate strains demonstrated an enhanced capacity to modulate the cell cycle under grazing pressure compared to fusiform strains, adapting more effectively to planktonic lifestyles under environmental stress (Song et al., 2020).

The endoplasmic reticulum (ER) serves as a crucial hub for protein synthesis and maturation, facilitating the post-translational modification, folding, and oligomerization of newly synthesized proteins (**Supplementary Figure 14**). Under grazing pressures, transcriptomic analysis of pt55_7 strains highlighted the role of CALR (calreticulin) in recognizing G1M9 glycoproteins in the ER. This process involves the inhibition of activity through hydrophobic entrapment and the promotion of folding in newly synthesized glycoproteins (Hirano et al., 2015). Concurrently, an increase in ER chaperone BiP, integral for luminal chaperones recognition, was noted, bolstering ER protein homeostasis (Kyeong and Lee, 2022). BiP, acting as a sentinel of ER integrity, targets aberrant proteins for proteasomal degradation and curtails aggregation, as anchoring the protein quality control system (Pobre et al., 2019). Chaperones such as DNAJA1 and CRYAA play a crucial role in marking aberrant proteins for ER-associated degradation (ERAD) (Shen et al., 2002). Proteomic scrutiny of pt55_7 strains under grazing pressures, accentuated the up-regulation of GANAB, facilitating deglycosylation processes (Gallo et al., 2018), and ERManI, responsible for mannose glycol-groups cleavage (Maki et al., 2022). The increased of HSP110 activity further substantiates the role of heat proteins in ER-associated degradation (Hrizo et al., 2007), while a decline in SKP1 activity implies attenuated proteasomal ubiquitination (Yoshida and Tanaka, 2010). This integrative analysis illuminates a nuanced regulation of protein synthesis and quality control in the pt55_7 strain’s ER, characterized by enhanced protein synthesis alongside proteasomal ubiquitination. This nuanced protein regulation under grazing pressures, varies across strains, highlighting the heterogeneity and complexity of their cellular responses.

In the Cell Cycle-yeast metabolic pathway (**Supplementary Figure 15**), specific genes (MCM2, ORC2, SMC2, YCS4) in the pt55_7 strain are down-regulated in response to grazing pressure, suggesting potential impediments in DNA replication and metaphase chromosome compaction. These intricate interactions among McM2-7 proteins, ORC components, and other essential factors like CDC6 and CDT1 are fundamental to DNA replication processes (Remus et al., 2009; Shibata and Dutta, 2020). Additionally, the collaboration between SMC2 and YCS4 establish a protein condensin complex pivotal for DNA morphology (Stray et al., 2005; Hassler et al., 2019). Among the strains studied, only pt55_7 exhibited significant alterations in the cell cycle, suggesting that the triradiate pt55_7 strain of *P.tricornutum* is more likely to modulate its cell cycle in response to grazing pressures than its fusiform strains.

#### 4.2.2 Fatty acid metabolism

Fatty acids play vital physiological roles in organisms, and the intricate alterations of fatty acid composition in *P.tricornutum* in response to grazing pressure are crucial in multi-omics analysis. The synthesis efficiency of long-chain fatty acids decreased at the transcriptome level and exhibited a slight increase at the proteome level, alongside an overall reduction in fatty acid abundance. This aligns with previous findings that environmental changes impact lipid profiles (Martino et al., 2011), with the triradiate strain showing greater resilience to grazing stress compared to the fusiform strain.

The study of fatty acid metabolism, especially in pt55_3, clarified the influence of grazing pressure on the biosynthesis, elongation, and degradation pathways. Within the fatty acid biosynthesis pathway (**Supplementary Figure 16**), a significant down-regulation of ACACA (Acetyl-CoA carboxylase) implies a reduction in ATP-dependent carboxylation of Acetyl-CoA to Malonyl-CoA under grazing pressure (Pereira et al., 2022). Enoyl ACP reductases, including FabI and its isomers (FabL, FabK, FabV, and InhA), play a crucial role in many microorganisms (Rana et al., 2020). A decline in the expression of FabI leads to a reduced processing of trans-2-Enoyl-[ACP] and Long-chain acyl-[ACP]. Prior to utilization within organisms, fatty acids require CoA conjugation (Ma et al., 2022). The down-regulation of FadD results in a slower conversion of Long-chain fatty acid to Long-chain acyl-CoA. Collectively, these findings highlight a decreased efficiency in fatty acid biosynthesis at the transcriptomic level due to grazing pressure. Within the fatty acid elongation metabolic pathway (**Supplementary Figure 17**), the diminished activity of HADH (3-hydroxyacyl-CoA dehydrogenase) during the processing of fatty acids containing between 4 and 16 carbon atoms suggests a suppressed oxidation reaction, consequently hindering fatty acid elongation at the proteomic level. Within the degradation pathway (**Supplementary Figure 18**), a decrease in the activities of HADH and ACADS results in a reduction of fatty acid dehydrogenation during the process of carbon chain reduction. Notably, the expression of ACAT, which is crucial in the terminal cleavage reactions of fatty acid degradation, is increased. Simultaneously, a down-regulated lncRNA, MSTRG.9570, has been found to be associated with ACAT modulation. Taken together, these findings suggest that grazing pressure hampers fatty acid oxidation while enhancing terminal cleavage reactions in degradation.

Collectively, omics analyses reveal that grazing pressures on *P.tricornutum* impede its fatty acid synthesis efficiency, favoring degradation processes, leading to a significant reduction in fatty acid quantity, which is in line with previous findings (Li and Ismar, 2018). At the transcriptomic level, the strains pt52_A and pt52_B exhibit enhanced fatty acid synthesis efficiencies, with variable reductions in elongation and degradation processes,not mirrored at the proteome and metabolome levels. Consequently, under grazing stress, both strains appear to favor the synthesis of short-chain fatty acids. In contrast, the pt55_7 strain exhibits only a modest proteomic increase in fatty acid synthesis, indicating distinct response patterns between fusiform (pt52_A, pt52_B, pt55_3) and triradiate (pt55_7) strains under grazing pressures.

#### 4.2.3 Signal response

Cells respond to environmental stress by undergoing complex signal transduction and regulatory actions. In the presence of grazing stress, *P.tricornutum* regulates Ca^2+^ levels through a variety of sensor proteins, resulting in changes at both the transcriptome and proteome levels.

Within the metabolic pathway of plant-pathogen interaction (**Supplementary Figure 19**), under grazing pressure, an up-regulation of CaMCML (Ca^2+^ calmodulin-like protein) in pt52_A strains suggests the translation of intracellular Ca^2+^ fluctuations into downstream signals through numerous sensor proteins, facilitating a defense against diverse stressors (Melo-Braga et al., 2012). This regulation could potentially lead to stomatal closure and increase the production of nitric oxide (NO), a molecule that is considered to be a sentinel against external threats and is thus crucial for plant defense (Robbins and Cowen, 2023). HSP90B, a universally conserved molecular chaperone, regulates the structure and function of numerous client proteins, many of which act as crucial signal transduction nodes (Kumar and Ohri, 2023). Intriguingly, under grazing stress, pt52_A strains manifest a down-regulation of HSP90B, potentially in response to stressors like the Hypersensitive Reaction (HR). Conversely, in the pt55_3 strain, an opposite trend is observed, where up-regulation of HSP90B may enhance signal transduction pathways to counteract stress.

In the Ca^2+^ signaling pathway (**Supplementary Figure 20**), the transcriptomic up-regulation of SPHK (sphingosine kinase) in pt52_A strains promotes sphingosine phosphorylation, which results in 1-phosphate sphingosine. Its equilibrium with ceramide plays a significant role in influencing sphingolipid dynamics (Adams et al., 2020; Smith et al., 2023). Moreover, with the up-regulation of CALM (calmodulin), Ca^2+^ modulates the MAPK signaling cascade, thus governing cellular processes including contraction, metabolism, and proliferation. The proteome of pt55_3 exhibits a remarkable up-regulation of PPIF (peptidyl-prolyl isomerase F (cyclophilin D)), which leads to compromised mitochondrial stability and functionality under Ca^2+^ stress (Gainutdinov et al., 2015). Consequently, it is apparent that under the influence of Ca^2+^, both pt52_A and pt55_3 strains promote cell apoptosis at the transcriptomic level; proteomic evidence simultaneously suggests impaired mitochondrial function. This observation provides a coherent explanation for the diminished cellular efficacy observed in the Ca^2+^ signaling pathway under grazing pressure in these strains, while other strains appear to remain relatively unaffected. In response to grazing pressure, intricate regulatory modifications across signaling pathways are discernible, emphasizing the dynamic predator-prey relationship between the grazers and *P.tricornutum*.

### 4.3 Weighted gene co-expression network analysis

Multi-Omics Insights into *P.tricornutum*’s Adaptation Under Grazing Stress Utilizing Weighted Gene Co-expression Network Analysis (WGCNA), core genes were correlated with transcriptomic, proteomic, and metabolomic datasets, constructing a multi-omics co-expression network pertinent to the observed phenotype (Li and Ismar, 2018). This analysis underscored the morphological modifications in the pt52_B and pt55_7 strains of *P.tricornutum* under grazing stress.

In pt52_B strain, phenotypic traits like total cell count, growth rate, and specific morphological variants of *P.tricornutum* were linked to metabolic routes such as Glycolysis/Gluconeogenesis (**Supplementary Figure 21**) and Carbon fixation in photosynthetic organisms (**Supplementary Figure 22**) at both the transcriptomic and proteomic levels. Notably, down-regulation of PGAM (2,3-bisphosphoglycerate-dependent phosphoglycerate mutase) inhibits the interconversion between 3-phosphoglyceric and 2-phosphoglyceric acid, highlighting PGAM’s pivotal role in metabolic flux and redox balance, as supported by its modulation of mitochondrial ROS and activation of the pentose phosphate pathway (Mikawa et al., 2020). Concurrently, up-regulation of GAPDH (aldehyde 3-phosphate dehydrogenase (phosphorylating)) and PGK (phosphoglycerate) underscores their importance in the carbon flux during Glycolysis/Gluconeogenesis, influencing cellular growth dynamics under grazing stress. Earlier work has similarly indicated such morphological adaptations in Phaeocystis globose in response to grazing, spotlighting the universality of this response (Yang et al., 2023).

For pt55_7, a correlation was found between phenotypic measures and the down-regulation of POLA1 (DNA polymerase alpha subunit A) at the transcriptomic level. Given that DNA polymerase alpha orchestrates replication initiation, the diminished activity of POLA1 hints at perturbed DNA replication dynamics (Begemann et al., 2022). In light of our multi-omics findings, this suggests that the triradiate *P.tricornutum*’s pt55_7 strain employs cellular replication regulation as an adaptive mechanism under grazing stress.

In strains pt52_A, pt55_3 and pt55_7, phenotypic alterations, particularly in fatty acid profiles, showed correlations across all omics layers. A collective downtrend in fatty acid synthesis emerged, intimating a reduction in the overall fatty acid pool. This hints at a strategic recalibration by *P.tricornutum* under grazing stress, aligning with the notion that modulation of fatty acid profiles is a crucial defense mechanism for plankton against predators. Such adjustments, mirroring changes in environmental pressures, are evident in other marine phytoplankton, reinforcing the idea that shifts in fatty acid content are universal stress markers in phytoplankton under grazer-induced stress (Antacli et al., 2021; Yang et al., 2023).

## Data availability statement

The datasets presented in this study can be found in online repositories. The names of the repository/repositories and accession number(s) can be found below: https://www.ncbi.nlm.nih.gov/, PRJNA1008380 http://www.proteomexchange.org/, PXD044954 https://www.ebi.ac.uk/metabolights/, MTBLS8485.

## Author contributions

CL: Writing – original draft, Writing – review & editing, Data curation, Formal analysis. LL: Writing – review & editing, Writing – original draft. SY: Writing – review & editing, Data curation, Formal analysis. MW: Writing – review & editing, Investigation. HZ: Writing – review & editing, Investigation. SL: Writing – original draft, Writing – review & editing, Data curation, Formal analysis, Investigation, Funding acquisition.
