# Supplementary material for: Multi-omic insights into the cellular response of Phaeodactylum tricornutum (Bacillariophyta) strains under grazing pressure
Source: Front Plant Sci. 2024 Jan 8;14:1308085. doi: 10.3389/fpls.2023.1308085 (PMC10801743; doi:10.3389/fpls.2023.1308085)
Supplement: Supplementary file 1 [file DataSheet_1.pdf]

Supplementary Material

1 Supplementary Tables

Table 2 RNASeq Map statistics

Sample	Clean_Reads	Total_Mapped	Multiple_Mapped	Uniquely_Mapped
pt52_A_c	98856954	86451437 (87.45%)	2117169 (2.45%)	84334268 (97.55%)
pt52_A_g	109112418	91420200 (83.79%)	2901106 (3.17%)	88519094 (96.83%)
pt52_B_c	99613970	79366612 (79.67%)	2225549 (2.80%)	77141063 (97.20%)
pt52_B_g	100799184	80137281 (79.50%)	2491966 (3.11%)	77645315 (96.89%)
pt55_3_c	96171702	82147879 (85.42%)	2663550 (3.24%)	79484329 (96.76%)
pt55_3_g	94442576	68702753 (72.75%)	2234617 (3.25%)	66468136 (96.75%)
pt55_7_c	107697388	85668197 (79.55%)	2059279 (2.40%)	83608918 (97.60%)
pt55_7_g	103032006	77736148 (75.45%)	1412496 (1.82%)	76323652 (98.18%)
pt55_8_c	101134866	92345055 (91.31%)	1495004 (1.62%)	90850051 (98.38%)

Note:

Clean Reads: The aggregate count of sequences utilized for alignment; **Total Mapped:** The cumulative count of sequences aligned to the reference genome; **Multiple Mapped:** The aggregate count of sequences that align to multiple genomic locations; **Uniquely Mapped:** The total sequences that align exclusively to a single genomic position.

Table 3 Phenotypic core genes

LncRNA Gene ID	mRNA Gene ID	mRNA KEGG ID
MSTRG.10215	Phatr3_J45354	K11270
MSTRG.10215	Phatr3_J26970	K17871
MSTRG.10215	Phatr3_J11823	K11252
MSTRG.10215	Phatr3_J468	K02542
MSTRG.10215	Phatr3_J26952	K01772
MSTRG.10215	Phatr3_J26925	K08819
MSTRG.10215	Phatr3_J26948	K01280
MSTRG.10215	Phatr3_J34976	K00799
MSTRG.10215	Phatr3_J11703	K14555
MSTRG.10215	Phatr3_J26980	K09493

MSTRG.10215	Phatr3_J11735	K03063
MSTRG.10215	Phatr3_J45316	K20184
MSTRG.10215	Phatr3_J26975	K03253
MSTRG.10215	Phatr3_J34984	K23309
MSTRG.10215	Phatr3_J35057	K01609
MSTRG.10215	Phatr3_J26934	K00033
MSTRG.10215	Phatr3_J35064	K18587
MSTRG.10215	Phatr3_J11634	K03978
MSTRG.10215	Phatr3_J35063	K11086
MSTRG.10215	Phatr3_J11722	K01866
MSTRG.10215	Phatr3_J41928	K05609
MSTRG.10215	Phatr3_J34971	K11254
MSTRG.10215	Phatr3_J26921	K01900
MSTRG.10215	Phatr3_J51837	K03676
MSTRG.10215	Phatr3_EG02095	K14838
MSTRG.11820	Phatr3_EG02359	K01597
MSTRG.11820	Phatr3_Jdraft1682	K13950
MSTRG.11820	Phatr3_EG02363	K00006
MSTRG.11820	Phatr3_EG02362	K02320
MSTRG.11820	Phatr3_Jdraft477	K02978
MSTRG.11820	Phatr3_Jdraft116	K08869
MSTRG.1750	Phatr3_J8132	K12833
MSTRG.1750	Phatr3_J13382	K01807
MSTRG.1750	Phatr3_J28219	K03061
MSTRG.1750	Phatr3_J28202	K02729
MSTRG.1750	Phatr3_J46780	K14709
MSTRG.1750	Phatr3_J13424	K03686
MSTRG.1750	Phatr3_J46726	K04648
MSTRG.1750	Phatr3_J52110	K14286
MSTRG.1750	Phatr3_J46736	K18477
MSTRG.1750	Phatr3_J46735	K02889
MSTRG.1750	Phatr3_J46782	K10765
MSTRG.1750	Phatr3_J13281	K07565

MSTRG.1750	Phatr3_J46790	K17408
MSTRG.1750	Phatr3_J54642	K13525
MSTRG.1750	Phatr3_J13538	K15116
MSTRG.1750	Phatr3_J36789	K23966
MSTRG.1750	Phatr3_J21073	K02975
MSTRG.1750	Phatr3_J46764	K13123
MSTRG.1750	Phatr3_J46785	K01784
MSTRG.1750	Phatr3_J8230	K04078
MSTRG.1750	Phatr3_J36821	K00006
MSTRG.1750	Phatr3_J13360	K11094
MSTRG.1750	Phatr3_J46740	K03004
MSTRG.1750	Phatr3_J28222	K00616
MSTRG.1750	Phatr3_J13428	K14553
MSTRG.1751	Phatr3_J8132	K12833
MSTRG.1751	Phatr3_J13382	K01807
MSTRG.1751	Phatr3_J28219	K03061
MSTRG.1751	Phatr3_J28202	K02729
MSTRG.1751	Phatr3_J46780	K14709
MSTRG.1751	Phatr3_J13424	K03686
MSTRG.1751	Phatr3_J46726	K04648
MSTRG.1751	Phatr3_J52110	K14286
MSTRG.1751	Phatr3_J46736	K18477
MSTRG.1751	Phatr3_J46735	K02889
MSTRG.1751	Phatr3_J46782	K10765
MSTRG.1751	Phatr3_J13281	K07565
MSTRG.1751	Phatr3_J46790	K17408
MSTRG.1751	Phatr3_J54642	K13525
MSTRG.1751	Phatr3_J13538	K15116
MSTRG.1751	Phatr3_J36789	K23966
MSTRG.1751	Phatr3_J21073	K02975
MSTRG.1751	Phatr3_J46764	K13123
MSTRG.1751	Phatr3_J46785	K01784
MSTRG.1751	Phatr3_J8230	K04078

MSTRG.1751	Phatr3_J36821	K00006
MSTRG.1751	Phatr3_J13360	K11094
MSTRG.1751	Phatr3_J46740	K03004
MSTRG.1751	Phatr3_J28222	K00616
MSTRG.1751	Phatr3_J13428	K14553
MSTRG.2215	Phatr3_J47120	K00784
MSTRG.2215	Phatr3_J47099	K12275
MSTRG.2215	Phatr3_J13622	K03028
MSTRG.2215	Phatr3_J7342	K22071
MSTRG.2215	Phatr3_J47116	K03023
MSTRG.2215	Phatr3_J52173	K08741
MSTRG.2215	Phatr3_J47090	K00275
MSTRG.2215	Phatr3_J37201	K15637
MSTRG.2215	Phatr3_J3251	K11323
MSTRG.2215	Phatr3_J13777	K01303
MSTRG.2215	Phatr3_J21354	K02259
MSTRG.2215	Phatr3_J47113	K13143
MSTRG.2215	Phatr3_J37260	K16075
MSTRG.2215	Phatr3_J47119	K10570
MSTRG.2215	Phatr3_J28482	K14403
MSTRG.2215	Phatr3_J37234	K05307
MSTRG.2215	Phatr3_J13581	K03164
MSTRG.2215	Phatr3_J47127	K10885
MSTRG.2529	Phatr3_J54726	K17339
MSTRG.2529	Phatr3_EG02303	K10589
MSTRG.2529	Phatr3_EG02525	K03715
MSTRG.2529	Phatr3_J13951	K00262
MSTRG.2529	Phatr3_J37520	K10903
MSTRG.2529	Phatr3_J42126	K10949
MSTRG.2529	Phatr3_J47297	K07766
MSTRG.2529	Phatr3_J14125	K03715
MSTRG.2529	Phatr3_J47327	K07195
MSTRG.2529	Phatr3_J47345	K06173

MSTRG.2529	Phatr3_J13855	K06182
MSTRG.2529	Phatr3_J21535	K07893
MSTRG.2529	Phatr3_J28652	K03644
MSTRG.2529	Phatr3_J21513	K01873
MSTRG.2529	Phatr3_J21548	K05658
MSTRG.2529	Phatr3_J47314	K16569
MSTRG.2529	Phatr3_J42129	K22182
MSTRG.2775	Phatr3_J47492	K17768
MSTRG.2775	Phatr3_J37723	K02328
MSTRG.2775	Phatr3_J54751	K17065
MSTRG.2775	Phatr3_J47510	K05275
MSTRG.2775	Phatr3_J14084	K13119
MSTRG.2775	Phatr3_J28797	K00507
MSTRG.2775	Phatr3_J21682	K15449
MSTRG.2775	Phatr3_EG02304	K15446
MSTRG.2775	Phatr3_J21667	K18423
MSTRG.2775	Phatr3_J47496	K12199
MSTRG.2775	Phatr3_J21659	K02912
MSTRG.4179	Phatr3_J29606	K01173
MSTRG.4179	Phatr3_J15068	K00228
MSTRG.4179	Phatr3_J4674	K14058
MSTRG.4179	Phatr3_J38886	K03355
MSTRG.4179	Phatr3_J48430	K03107
MSTRG.4179	Phatr3_J38930	K12251
MSTRG.4179	Phatr3_J14927	K19054
MSTRG.4179	Phatr3_J14917	K00604
MSTRG.4179	Phatr3_J38893	K12189
MSTRG.4179	Phatr3_J15019	K15507
MSTRG.4179	Phatr3_J29561	K15498
MSTRG.4179	Phatr3_J38931	K15178
MSTRG.4179	Phatr3_J52412	K05016
MSTRG.4179	Phatr3_J22459	K12418
MSTRG.4179	Phatr3_J38891	K00817

MSTRG.4550	Phatr3_J22658	K11108
MSTRG.4550	Phatr3_J29793	K02493
MSTRG.4550	Phatr3_J22677	K04712
MSTRG.4550	Phatr3_J22666	K09498
MSTRG.4890	Phatr3_J32531	K03232
MSTRG.4890	Phatr3_J41721	K04077
MSTRG.4890	Phatr3_J43314	K22736
MSTRG.4890	Phatr3_J7634	K14819
MSTRG.4890	Phatr3_J43326	K15442
MSTRG.4890	Phatr3_J9929	K11884
MSTRG.4890	Phatr3_J9860	K11462
MSTRG.4890	Phatr3_J50701	K02973
MSTRG.5888	Phatr3_J30160	K02976
MSTRG.5888	Phatr3_J49185	K21891
MSTRG.5888	Phatr3_J49172	K17780
MSTRG.5888	Phatr3_J15709	K11097
MSTRG.5888	Phatr3_J42274	K20302
MSTRG.5888	Phatr3_J49146	K24939
MSTRG.5888	Phatr3_J30139	K07901
MSTRG.5888	Phatr3_J49167	K00383
MSTRG.5888	Phatr3_J30145	K01647
MSTRG.5891	Phatr3_J30160	K02976
MSTRG.5891	Phatr3_J49185	K21891
MSTRG.5891	Phatr3_J49172	K17780
MSTRG.5891	Phatr3_J15709	K11097
MSTRG.5891	Phatr3_J42274	K20302
MSTRG.5891	Phatr3_J49146	K24939
MSTRG.5891	Phatr3_J30139	K07901
MSTRG.5891	Phatr3_J49167	K00383
MSTRG.5891	Phatr3_J30145	K01647
MSTRG.6322	Phatr3_J49487	K14589
MSTRG.6322	Phatr3_EG02569	K03783
MSTRG.6322	Phatr3_J30389	K04649

MSTRG.6322	Phatr3_J49488	K08994
MSTRG.6322	Phatr3_J49527	K11274
MSTRG.6322	Phatr3_J40214	K13130
MSTRG.6322	Phatr3_J23247	K03841
MSTRG.6322	Phatr3_J16018	K03596
MSTRG.6322	Phatr3_J49471	K06199
MSTRG.6322	Phatr3_J23292	K00225
MSTRG.6322	Phatr3_J15917	K01758
MSTRG.6322	Phatr3_J15937	K11422
MSTRG.6322	Phatr3_J16008	K10418
MSTRG.6322	Phatr3_J49524	K00889
MSTRG.6322	Phatr3_J49469	K12829
MSTRG.6322	Phatr3_J55057	K02714
MSTRG.6322	Phatr3_J49507	K11491
MSTRG.6322	Phatr3_J49529	K24752
MSTRG.6322	Phatr3_J15863	K10908
MSTRG.6322	Phatr3_J30394	K12483
MSTRG.6322	Phatr3_EG02566	K11600
MSTRG.6322	Phatr3_EG02568	K19513
MSTRG.6372	Phatr3_J23314	K10393
MSTRG.6372	Phatr3_J49527	K11274
MSTRG.6372	Phatr3_J16018	K03596
MSTRG.6372	Phatr3_J51214	K00264
MSTRG.6372	Phatr3_J23292	K00225
MSTRG.6372	Phatr3_J15917	K01758
MSTRG.6372	Phatr3_J49533	K03301
MSTRG.6372	Phatr3_J49524	K00889
MSTRG.6372	Phatr3_J23306	K12585
MSTRG.6372	Phatr3_J49507	K11491
MSTRG.6372	Phatr3_J49529	K24752
MSTRG.6372	Phatr3_EG02566	K11600
MSTRG.6392	Phatr3_J49563	K12373
MSTRG.6392	Phatr3_J49572	K10875

MSTRG.6392	Phatr3_J30461	K00213
MSTRG.6392	Phatr3_J55067	K14801
MSTRG.6392	Phatr3_J40325	K17491
MSTRG.6392	Phatr3_J30446	K09496
MSTRG.6392	Phatr3_J49568	K14209
MSTRG.6392	Phatr3_J16125	K02924
MSTRG.6392	Phatr3_J23324	K02868
MSTRG.6672	Phatr3_J23429	K00602
MSTRG.6672	Phatr3_J40521	K08493
MSTRG.6672	Phatr3_J55091	K01669
MSTRG.6672	Phatr3_J49717	K07432
MSTRG.6672	Phatr3_J40517	K14209
MSTRG.6672	Phatr3_J52607	K06669
MSTRG.6672	Phatr3_J23444	K06207
MSTRG.6672	Phatr3_J16281	K03248
MSTRG.6721	Phatr3_J55102	K02293
MSTRG.6721	Phatr3_J40521	K08493
MSTRG.6721	Phatr3_J52619	K23887
MSTRG.6721	Phatr3_J40517	K14209
MSTRG.6721	Phatr3_J23467	K00140
MSTRG.6721	Phatr3_J23444	K06207
MSTRG.6721	Phatr3_J16281	K03248
MSTRG.6836	Phatr3_J49849	K15272
MSTRG.6836	Phatr3_J49836	K05292
MSTRG.6836	Phatr3_J30670	K02971
MSTRG.6836	Phatr3_EG02222	K00323
MSTRG.6836	Phatr3_J23547	K01881
MSTRG.6838	Phatr3_J49849	K15272
MSTRG.6838	Phatr3_J49836	K05292
MSTRG.6838	Phatr3_J30670	K02971
MSTRG.6838	Phatr3_EG02222	K00323
MSTRG.6838	Phatr3_J23547	K01881
MSTRG.7075	Phatr3_J23658	K03839

MSTRG.7075	Phatr3_J30786	K01805
MSTRG.7075	Phatr3_J40841	K07936
MSTRG.7183	Phatr3_J16499	K00641
MSTRG.7183	Phatr3_J50084	K11188
MSTRG.7183	Phatr3_EG02218	K01885
MSTRG.7183	Phatr3_J40956	K09500
MSTRG.7183	Phatr3_J40942	K10733
MSTRG.7183	Phatr3_J5685	K02727
MSTRG.7183	Phatr3_J16430	K03885
MSTRG.7183	Phatr3_J16421	K24175
MSTRG.7183	Phatr3_EG02219	K01867
MSTRG.7183	Phatr3_J16511	K02887
MSTRG.7183	Phatr3_J23717	K02641
MSTRG.7183	Phatr3_J50081	K08869
MSTRG.7183	Phatr3_J50075	K03321
MSTRG.7183	Phatr3_J40930	K01764
MSTRG.7183	Phatr3_J23691	K02957
MSTRG.7183	Phatr3_J50048	K12620
MSTRG.7214	Phatr3_J16499	K00641
MSTRG.7214	Phatr3_J50084	K11188
MSTRG.7214	Phatr3_EG02218	K01885
MSTRG.7214	Phatr3_J23748	K02979
MSTRG.7214	Phatr3_J40956	K09500
MSTRG.7214	Phatr3_J40942	K10733
MSTRG.7214	Phatr3_J16430	K03885
MSTRG.7214	Phatr3_J16511	K02887
MSTRG.7214	Phatr3_J23717	K02641
MSTRG.7214	Phatr3_J50081	K08869
MSTRG.7214	Phatr3_J50075	K03321
MSTRG.7214	Phatr3_J40930	K01764
MSTRG.7214	Phatr3_J16493	K12188
MSTRG.7664	Phatr3_J31156	K10413
MSTRG.7664	Phatr3_J16854	K03676

MSTRG.7664	Phatr3_J31140	K09550
MSTRG.7664	Phatr3_J51283	K12831
MSTRG.7664	Phatr3_J31133	K02151
MSTRG.7664	Phatr3_J16891	K12400
MSTRG.7664	Phatr3_J16843	K11253
MSTRG.7664	Phatr3_J24069	K01726
MSTRG.7664	Phatr3_J24006	K01410
MSTRG.7664	Phatr3_J2171	K02575
MSTRG.7664	Phatr3_J16840	K01726
MSTRG.7664	Phatr3_J16870	K00021
MSTRG.7664	Phatr3_J16859	K11412
MSTRG.8219	Phatr3_J33525	K04083
MSTRG.8219	Phatr3_J5873	K23563
MSTRG.8219	Phatr3_J33504	K11368
MSTRG.8219	Phatr3_J25840	K00323
MSTRG.8219	Phatr3_J44118	K15289
MSTRG.8219	Phatr3_J10362	K24083
MSTRG.8219	Phatr3_J18745	K00012
MSTRG.8219	Phatr3_J44171	K22755
MSTRG.8219	Phatr3_J33530	K18534
MSTRG.8219	Phatr3_J10208	K00855
MSTRG.8219	Phatr3_J41807	K00654
MSTRG.8219	Phatr3_J44136	K15032
MSTRG.8219	Phatr3_J10497	K07512
MSTRG.8219	Phatr3_J51630	K25304
MSTRG.8219	Phatr3_J44117	K03848
MSTRG.8219	Phatr3_J33493	K01868
MSTRG.8219	Phatr3_EG02519	K00852
MSTRG.8219	Phatr3_J44165	K06675
MSTRG.8219	Phatr3_J10196	K02922
MSTRG.8219	Phatr3_EG02394	K12604
MSTRG.8219	Phatr3_J3755	K13354
MSTRG.8219	Phatr3_J44146	K10735

MSTRG.8219	Phatr3_J33540	K12877
MSTRG.8449	Phatr3_J50465	K10696
MSTRG.8449	Phatr3_J6457	K13800
MSTRG.8449	Phatr3_J41450	K14411
MSTRG.8449	Phatr3_J41423	K01623
MSTRG.8449	Phatr3_J41470	K00002
MSTRG.8574	Phatr3_EG00041	K08678
MSTRG.8574	Phatr3_J42398	K00026
MSTRG.8574	Phatr3_J8024	K02890
MSTRG.8574	Phatr3_J17048	K17292
MSTRG.8574	Phatr3_J41570	K10257
MSTRG.8574	Phatr3_J24195	K01948
MSTRG.8574	Phatr3_J50559	K07556
MSTRG.8574	Phatr3_J17086	K01834
MSTRG.8574	Phatr3_J24223	K13985
MSTRG.8941	Phatr3_J44426	K12735
MSTRG.8941	Phatr3_J44454	K06897
MSTRG.8941	Phatr3_J44425	K03847
MSTRG.8941	Phatr3_J44459	K03259
MSTRG.8941	Phatr3_J3687	K07442
MSTRG.8941	Phatr3_J33839	K01834
MSTRG.8941	Phatr3_EG02636	K08860
MSTRG.8941	Phatr3_J50796	K03265
MSTRG.8941	Phatr3_J33864	K00993
MSTRG.8941	Phatr3_J10677	K17686
MSTRG.8941	Phatr3_J33914	K20300
MSTRG.8941	Phatr3_J44439	K03105
MSTRG.8941	Phatr3_J10824	K00559
MSTRG.9524	Phatr3_J34457	K21991
MSTRG.9524	Phatr3_J2217	K25193
MSTRG.9524	Phatr3_J34422	K03119
MSTRG.9524	Phatr3_J44905	K03849
MSTRG.9524	Phatr3_J11259	K02367

MSTRG.9524	Phatr3_EG02346	K22066
MSTRG.9524	Phatr3_J34391	K18172
MSTRG.9524	Phatr3_J26515	K00413
MSTRG.9524	Phatr3_J19438	K14539
MSTRG.9524	Phatr3_J44879	K14001
MSTRG.9524	Phatr3_EG02349	K02291
MSTRG.9524	Phatr3_J19413	K02923
MSTRG.9524	Phatr3_J1884	K01255
MSTRG.9524	Phatr3_J259	K12874
MSTRG.9524	Phatr3_J44902	K01914
MSTRG.9524	Phatr3_J44850	K01090
MSTRG.9570	Phatr3_J34457	K21991
MSTRG.9570	Phatr3_J34422	K03119
MSTRG.9570	Phatr3_J44905	K03849
MSTRG.9570	Phatr3_J44949	K00626
MSTRG.9570	Phatr3_J11403	K08867
MSTRG.9570	Phatr3_J19438	K14539
MSTRG.9570	Phatr3_J11363	K12795
MSTRG.9570	Phatr3_EG02345	K08292
MSTRG.9570	Phatr3_J44935	K22748
MSTRG.9570	Phatr3_J34493	K03267
MSTRG.9570	Phatr3_J2808	K09584
MSTRG.9570	Phatr3_J44902	K01914
MSTRG.9570	Phatr3_J54326	K03142
MSTRG.9705	Phatr3_J5424	K14785
MSTRG.9705	Phatr3_EG02355	K09272
MSTRG.9705	Phatr3_J5532	K19589
MSTRG.9705	Phatr3_J44969	K08869
MSTRG.9705	Phatr3_J11373	K03885
MSTRG.9705	Phatr3_J45009	K03131
MSTRG.9705	Phatr3_J19518	K01754
MSTRG.9705	Phatr3_J11198	K10393
MSTRG.9705	Phatr3_J34582	K01778

MSTRG.9705	Phatr3_J44981	K10563
MSTRG.9705	Phatr3_J26649	K03341
MSTRG.9705	Phatr3_J11305	K03965
MSTRG.9705	Phatr3_J34592	K01669
MSTRG.9705	Phatr3_J8185	K02929
MSTRG.9705	Phatr3_J34610	K03434
MSTRG.9705	Phatr3_J34538	K03237
MSTRG.9705	Phatr3_J34526	K01755

Table 5 KEGG pathway enrichment analysis of Proteomic

Pathway ID	KEGG ID	Control	Grazing	Log2FoldChange	Strains
map04110	K03083	1.247177083	0.806721093	-0.62852444121815	pt52_B
	K06670	1.59879169	0.819039698	-0.964976694699439	pt55_3
	K03094	1.031746749	0.631415186	-0.708428029295167	pt55_7
	K03083	1.016646329	1.590583967	0.645738652286047	pt55_7
map04146	K00624	1.153087836	0.755644212	-0.609723394783453	pt55_3
	K04564	0.872352629	1.663087648	0.930880868892113	pt55_3
	K07753	1.0413251	1.629799098	0.646273591780793	pt55_7
	K13348	0.811290264	1.27917976	0.656928935865247	pt55_7
	K01578	0.89154542	1.675673949	0.910361254742207	pt55_7
map04626	K04079	0.67160695	1.035053921	0.624016862675882	pt52_A
map04714	K15109	1.374849996	0.826226907	-0.734664272316364	pt52_A
	K11652	0.51852357	0.79339521	0.613630118340369	pt52_A
	K03939	1.15059812	0.559226842	-1.04087850343284	pt52_B
	K02267	1.236122631	0.715259947	-0.789282312411802	pt52_B
	K03941	0.751331014	1.144908961	0.607712323695332	pt55_3
	K02267	0.792445105	1.275343508	0.686502977529588	pt55_3
	K18162	0.766603918	1.364845255	0.832184111540265	pt55_3
	K03943	0.667755928	1.194988983	0.83960453339075	pt55_3
	K11652	0.479257135	1.603240874	1.74211938043525	pt55_3
	K07199	0.998675737	0.524170928	-0.929978981154119	pt55_7
	K03942	1.0065054	1.604093626	0.672403436626415	pt55_7
	K11652	0.506350829	3.368572011	2.73392792103245	pt55_7

map04020	K09565	0.823432309	1.290632576	0.648356382939535	pt55_3
map03018	K00850	0.681116895	1.122258482	0.72043067691411	pt52_A
	K12591	1.127763065	0.551137758	-1.03297912735737	pt52_A
	K12580	0.628319528	1.173397648	0.901121681116464	pt52_A
	K00850	0.636690346	0.975722209	0.615878576931743	pt52_B
	K00850	2.046994378	0.72265278	-1.50213260790351	pt55_3
	K12591	1.548652969	0.750897752	-1.04432551568433	pt55_3
	K12580	1.172273362	0.749926965	-0.644487027540342	pt55_3
	K12626	1.529844619	1.009526399	-0.599706492530827	pt55_3
	K03654	0.66616902	1.324957834	0.991986277777325	pt55_3
	K12591	1.504998521	0.869894324	-0.790850012019788	pt55_7
	K12606	1.358825015	0.827183668	-0.716080075731092	pt55_7
	K03654	0.818871645	1.280499029	0.644996921009042	pt55_7
	K12625	0.938152409	1.504688737	0.681570857204844	pt55_7
map03050	K03029	1.14703793	0.76047263	-0.59294486784831	pt55_3
	K03033	0.903096948	1.408504961	0.641211869955289	pt55_7
	K02738	0.913260474	1.511756907	0.727127871067036	pt55_7
	K03030	0.933540057	1.714733139	0.877200237008292	pt55_7
map03060	K09540	0.782265686	1.400250205	0.839954051441407	pt52_A
	K12275	0.761941412	1.155797594	0.601136797959164	pt52_A
	K03100	1.583628024	1.035661669	-0.612680723603363	pt52_B
	K09540	1.149272846	0.737449323	-0.640105528437941	pt55_3
	K03105	0.801585629	1.230004655	0.617735226291408	pt55_3
	K12947	0.642961954	1.044166923	0.699547087244376	pt55_3
	K03217	0.851506172	0.476530579	-0.837448191403282	pt55_7
	K12948	0.858037962	1.296158934	0.59512924698978	pt55_7
	K03105	0.879195717	1.38657757	0.657272064046815	pt55_7
map04141	K09540	0.782265686	1.400250205	0.839954051441407	pt52_A
	K13993	2.782906988	1.124293313	-1.3075742257048	pt52_A
	K12275	0.761941412	1.155797594	0.601136797959164	pt52_A
	K11519	0.606781461	1.2119697	0.998104718798943	pt52_A
	K04079	0.67160695	1.035053921	0.624016862675882	pt52_A
	K10839	1.353516322	0.773724158	-0.806821060259171	pt52_B

	K11519	0.646961364	1.087070421	0.7486939387992	pt52_B
	K04554	1.800460889	0.944351296	-0.930970718097615	pt55_3
	K14016	1.084831525	0.624652339	-0.796345648526468	pt55_3
	K09540	1.149272846	0.737449323	-0.640105528437941	pt55_3
	K10575	0.844603668	1.283560546	0.603804932237405	pt55_3
	K13993	0.542538657	0.97558917	0.846547806059685	pt55_3
	K06689	0.687687294	1.236877741	0.8468783081211	pt55_3
	K03094	1.031746749	0.631415186	-0.708428029295167	pt55_7
	K09562	0.874333977	1.332609377	0.607997581848863	pt55_7
	K01230	1.213026004	1.924431462	0.665821812799019	pt55_7
	K05546	0.923479388	1.506490107	0.706039533595112	pt55_7
	K09523	0.950900612	2.0225001	1.08877331002326	pt55_7
map00010	K00850	0.681116895	1.122258482	0.72043067691411	pt52_A
	K01623	1.27611351	0.687574535	-0.892168642160696	pt52_B
	K00850	0.636690346	0.975722209	0.615878576931743	pt52_B
	K00850	2.046994378	0.72265278	-1.50213260790351	pt55_3
	K00134	1.111465271	0.739528714	-0.587784801750161	pt55_3
	K03841	1.177011948	0.622242202	-0.919580815149642	pt55_7
	K01834	1.305186798	0.809198311	-0.689691084902749	pt55_7
	K00927	1.377105772	0.860823721	-0.677849634072602	pt55_7
	K00162	0.852126039	0.563941587	-0.595521101707901	pt55_7
	K00134	1.02179164	1.728020421	0.758019229411658	pt55_7
	K01810	0.737523988	1.620015201	1.13524547125369	pt55_7
map00020	K00162	0.852126039	0.563941587	-0.595521101707901	pt55_7
map00030	K00850	0.681116895	1.122258482	0.72043067691411	pt52_A
	K01623	1.27611351	0.687574535	-0.892168642160696	pt52_B
	K00850	0.636690346	0.975722209	0.615878576931743	pt52_B
	K00850	2.046994378	0.72265278	-1.50213260790351	pt55_3
	K03841	1.177011948	0.622242202	-0.919580815149642	pt55_7
	K00616	1.352932764	0.785359082	-0.784665803858389	pt55_7
	K01807	1.434559443	0.94658498	-0.599803814450694	pt55_7
	K00948	0.991657511	1.508523742	0.605223554362091	pt55_7
	K01810	0.737523988	1.620015201	1.13524547125369	pt55_7

map00051	K00850	0.681116895	1.122258482	0.72043067691411	pt52_A
	K01623	1.27611351	0.687574535	-0.892168642160696	pt52_B
	K00850	0.636690346	0.975722209	0.615878576931743	pt52_B
	K00850	2.046994378	0.72265278	-1.50213260790351	pt55_3
	K03841	1.177011948	0.622242202	-0.919580815149642	pt55_7
map00052	K00850	0.681116895	1.122258482	0.72043067691411	pt52_A
	K00850	0.636690346	0.975722209	0.615878576931743	pt52_B
	K00850	2.046994378	0.72265278	-1.50213260790351	pt55_3
map00053	K00434	0.970525528	1.623404191	0.742184176860024	pt55_3
	K00434	1.255113225	0.713469017	-0.814894830284862	pt55_7
	K00225	0.742190793	1.721548069	1.21384445438515	pt55_7
map00061	K03921	0.974918623	1.473886503	0.59627172793089	pt55_7
	K09458	0.804709583	1.499771489	0.898202584601143	pt55_7
map00062	K00022	1.936338537	0.852040932	-1.1843365611956	pt55_3
map00071	K00022	1.936338537	0.852040932	-1.1843365611956	pt55_3
	K00248	1.43510003	0.718096541	-0.998901580744342	pt55_3
	K00626	0.752619978	1.220153938	0.697069683935568	pt55_3
	K00248	0.944502465	1.60969041	0.769156776381336	pt55_7
map00190	K08738	0.550259266	1.06837853	0.957239449961664	pt52_A
	K02151	1.187696841	0.790144284	-0.587978609841827	pt52_A
	K01507	0.612965953	1.368134178	1.15833088072524	pt52_A
	K03939	1.15059812	0.559226842	-1.04087850343284	pt52_B
	K02152	1.616716021	0.823157594	-0.973825722307937	pt52_B
	K02267	1.236122631	0.715259947	-0.789282312411802	pt52_B
	K08738	0.49277363	0.848900204	0.784669906451857	pt52_B
	K03953	0.768355804	1.165105282	0.600613884003827	pt55_3
	K03941	0.751331014	1.144908961	0.607712323695332	pt55_3
	K02267	0.792445105	1.275343508	0.686502977529588	pt55_3
	K03943	0.667755928	1.194988983	0.83960453339075	pt55_3
	K08738	0.823108436	1.54445275	0.907941326884655	pt55_3
	K02152	0.841140335	0.48483494	-0.79485284816461	pt55_7
	K03942	1.0065054	1.604093626	0.672403436626415	pt55_7
map00195	K08906	0.833339933	1.31881069	0.662260467153219	pt52_A

	K08906	0.856494819	1.334510653	0.63979439756844	pt52_B
	K08901	1.236179101	0.772678607	-0.677947420853675	pt55_3
	K02636	1.264573681	0.792729434	-0.67375064802969	pt55_3
	K02641	1.009359024	1.546784571	0.615832853219709	pt55_3
	K02641	1.111149127	0.600804908	-0.887083949334208	pt55_7
	K08906	1.217343464	0.736200623	-0.725565394226366	pt55_7
	K02716	0.985610231	0.59955541	-0.717124140289182	pt55_7
	K08901	0.878488737	0.568063155	-0.628972457712928	pt55_7
	K02636	0.854138742	2.184331821	1.35464969450844	pt55_7
map00220	K00262	1.423186519	2.20491771	0.631600064280177	pt55_3
map00230	K00939	1.128489667	0.658758047	-0.776572623247723	pt55_7
	K00944	0.823820389	0.507635117	-0.69853795923203	pt55_7
	K00942	0.822113655	0.507648927	-0.695506734267336	pt55_7
	K12304	0.775039493	1.171302512	0.595771996152988	pt55_7
	K00948	0.991657511	1.508523742	0.605223554362091	pt55_7
	K01487	0.852790197	1.605618107	0.912866031979949	pt55_7
map00250	K00262	1.423186519	2.20491771	0.631600064280177	pt55_3
map00260	K02437	1.005283043	0.570829429	-0.816470138221402	pt52_A
	K00600	1.133154618	0.683070002	-0.730239388517336	pt55_3
	K01834	1.305186798	0.809198311	-0.689691084902749	pt55_7
	K00600	0.856502501	1.493767643	0.802426388923712	pt55_7
	K00928	0.914917939	1.71192751	0.903907356608559	pt55_7
map00280	K00140	1.611923233	0.579045883	-1.47703346311013	pt55_3
	K00022	1.936338537	0.852040932	-1.1843365611956	pt55_3
	K00248	1.43510003	0.718096541	-0.998901580744342	pt55_3
	K00253	1.450182655	0.8311686	-0.803021566051337	pt55_3
	K01968	1.414509084	0.91264062	-0.632182669703957	pt55_3
	K00826	1.019024044	1.541220491	0.596885180115129	pt55_3
	K00626	0.752619978	1.220153938	0.697069683935568	pt55_3
	K05605	0.951558388	1.552858197	0.706562004389667	pt55_7
	K00248	0.944502465	1.60969041	0.769156776381336	pt55_7
	K00253	0.831730096	1.637161025	0.977008885132912	pt55_7
	K00140	0.85571898	2.125401565	1.31252644924726	pt55_7

map00480	K00434	0.970525528	1.623404191	0.742184176860024	pt55_3
	K00434	1.255113225	0.713469017	-0.814894830284862	pt55_7
	K00797	0.84808206	1.331787629	0.651088272802541	pt55_7
map00520	K00326	0.977689927	1.562171535	0.67610398453706	pt55_7
	K01810	0.737523988	1.620015201	1.13524547125369	pt55_7
map00562	K00140	1.611923233	0.579045883	-1.47703346311013	pt55_3
	K00140	0.85571898	2.125401565	1.31252644924726	pt55_7
map00564	K13535	0.738354758	0.318116622	-1.21475839977393	pt52_B
	K13535	0.945850025	0.360152254	-1.39300451437437	pt55_3
	K00894	0.884049408	1.33331504	0.592818797616575	pt55_7
	K00006	1.091728732	1.686490365	0.627409650189827	pt55_7
	K13535	1.08980534	3.28692914	1.59266989026791	pt55_7
map00620	K00626	0.752619978	1.220153938	0.697069683935568	pt55_3
	K01759	0.925694635	1.734426519	0.905850454620948	pt55_3
	K00162	0.852126039	0.563941587	-0.595521101707901	pt55_7
map00630	K02437	1.005283043	0.570829429	-0.816470138221402	pt52_A
	K00600	1.133154618	0.683070002	-0.730239388517336	pt55_3
	K00626	0.752619978	1.220153938	0.697069683935568	pt55_3
	K01455	0.94408466	1.565934411	0.730035643664301	pt55_7
	K00600	0.856502501	1.493767643	0.802426388923712	pt55_7
map00640	K00140	1.611923233	0.579045883	-1.47703346311013	pt55_3
	K00248	1.43510003	0.718096541	-0.998901580744342	pt55_3
	K05605	0.951558388	1.552858197	0.706562004389667	pt55_7
	K00248	0.944502465	1.60969041	0.769156776381336	pt55_7
	K01578	0.89154542	1.675673949	0.910361254742207	pt55_7
	K00140	0.85571898	2.125401565	1.31252644924726	pt55_7
map00680	K00850	0.681116895	1.122258482	0.72043067691411	pt52_A
	K01623	1.27611351	0.687574535	-0.892168642160696	pt52_B
	K00850	0.636690346	0.975722209	0.615878576931743	pt52_B
	K00850	2.046994378	0.72265278	-1.50213260790351	pt55_3
	K00600	1.133154618	0.683070002	-0.730239388517336	pt55_3
	K03841	1.177011948	0.622242202	-0.919580815149642	pt55_7
	K01834	1.305186798	0.809198311	-0.689691084902749	pt55_7

	K00600	0.856502501	1.493767643	0.802426388923712	pt55_7
map00710	K01623	1.27611351	0.687574535	-0.892168642160696	pt52_B
	K00134	1.111465271	0.739528714	-0.587784801750161	pt55_3
	K03841	1.177011948	0.622242202	-0.919580815149642	pt55_7
	K00927	1.377105772	0.860823721	-0.677849634072602	pt55_7
	K01807	1.434559443	0.94658498	-0.599803814450694	pt55_7
	K00134	1.02179164	1.728020421	0.758019229411658	pt55_7
map00790	K03639	1.620129883	0.956288018	-0.76059237192889	pt52_A
	K01307	1.57193969	0.850961155	-0.885380685743356	pt55_3
	K13998	1.428718107	0.855053165	-0.740635262276338	pt55_3
map00860	K01749	0.76676402	1.242266426	0.696120071438441	pt52_B
	K19054	0.990854943	1.782056039	0.84679693094946	pt52_B
	K01719	1.39703241	0.831861847	-0.747949635621983	pt55_3
	K19054	0.98944199	0.546524944	-0.85632778194681	pt55_7
	K00798	1.110898103	0.720117646	-0.625421966649125	pt55_7
	K01764	0.737188389	1.126121746	0.611257553971841	pt55_7
map00900	K03527	1.816830316	1.185678975	-0.615710233864955	pt52_A
	K03527	1.968748963	0.940720531	-1.06544106656246	pt52_B
	K03527	0.441885985	0.715471713	0.695220552297464	pt55_3
	K00626	0.752619978	1.220153938	0.697069683935568	pt55_3
	K13789	1.41452668	0.791396468	-0.837846856788745	pt55_7
	K00991	0.790863976	1.301471891	0.718642668092901	pt55_7
map00910	K01725	1.144134905	0.676328382	-0.758461367129894	pt55_3
	K00262	1.423186519	2.20491771	0.631600064280177	pt55_3
	K01725	1.051849911	1.744443543	0.729838047007609	pt55_7
	K01455	0.94408466	1.565934411	0.730035643664301	pt55_7
	K02575	0.535496968	0.948906189	0.825387059929189	pt55_7
map04111	K06674	1.275509174	0.771827089	-0.72472369024081	pt52_A
	K03094	1.031746749	0.631415186	-0.708428029295167	pt55_7

Table 6 KEGG pathway enrichment analysis of Metabolomic

Pathway ID	Compounds	Control	Grazing	Log2FoldChange	Strains

pti00970	C00407|L-Isoleucine|1	231044613.9	494270087.2	1.09712814053324	pt52_B
	C00082|L-Tyrosine|1	24373739.05	80178758.72	1.71789249818785	pt55_3
	C00407|L-Isoleucine|1	382983783.4	1385254435	1.8547957754412	pt55_3
	C00407|L-Isoleucine|1	54467655.64	290272477.3	2.41393610840039	pt55_7
	C00079|L-Phenylalanine|1	20288168.98	123978589.2	2.61138042067444	pt55_7
	C00082|L-Tyrosine|1	9781543.222	21037425.83	1.10482418469978	pt55_7
pti00230	C00147|Adenine|1	18476231.76	46494730.73	1.3313966761344	pt52_A
	C00212|Adenosine|1	77256476.22	246695364.7	1.67500282774815	pt52_A
	C00242|Guanine|1	1852894.06	6523336.641	1.81582968447622	pt52_A
	C00147|Adenine|1	46724736.45	22889766.64	-1.02948377161121	pt55_7
pti00260	C00300|Creatine|1	2278934.218	5223037.917	1.19652989567471	pt52_B
	C00114|Choline|1	492663090.2	1159945351	1.23538354042385	pt55_3
	C00300|Creatine|1	324930819.6	73538576.91	-2.14355942586252	pt55_3
pti00280	C00407|L-Isoleucine|1	231044613.9	494270087.2	1.09712814053324	pt52_B
	C00407|L-Isoleucine|1	382983783.4	1385254435	1.8547957754412	pt55_3
	C00407|L-Isoleucine|1	54467655.64	290272477.3	2.41393610840039	pt55_7
pti00480	C00315|Spermidine|1	1916452.155	849451.018	-1.17383531660996	pt55_7
pti00564	C06771|Triethanolamine|1	29057415.44	5179273.614	-2.48808470486864	pt52_A
	C06771|Triethanolamine|1	7941284.095	3707618.164	-1.09887963507133	pt52_B
	C00114|Choline|1	492663090.2	1159945351	1.23538354042385	pt55_3
pti00600	C12144|Phytosphingosine|1	11355288.93	26273905.68	1.21026626119145	pt52_A
	C12144|Phytosphingosine|1	41907032.39	105075868.5	1.32616711387301	pt52_B
pti00860	C03114|Dimethylbenzimidazole|1	32795092.4	11158250.64	-1.55536907652648	pt52_A
	C05769|Coproporphyrin I|1	399057648.4	125678013.7	-1.66686489016671	pt55_3
	C03114|Dimethylbenzimidazole|1	8786426.984	39061917.37	2.15241425079516	pt55_7
	C05769|Coproporphyrin I|1	41072245.28	16567222.25	-1.30983208353387	pt55_7
pti00900	C01126|(2E,6E)-Farnesol|1	2595335.017	13567866.7	2.38620121619472	pt55_3
	C01126|(2E,6E)-Farnesol|1	4976339.41	11705629.93	1.23404578117987	pt55_7

Table 7 KEGG pathway enrichment analysis of Transcriptomic

Pathway ID	Strains	KEGG ID	Log2FoldChange
map00010	pt52_A	K00134	2.907928424

	pt52_A	K00129	1.316720765
	pt52_A	K00382	1.071539594
	pt52_A	K01803	-1.091345651
map00061	pt52_A	K09458	3.50796419
	pt52_A	K00645	2.973613173
	pt52_A	K11262	1.403861653
map00062	pt52_A	K07509	-1.747655733
	pt52_A	K07508	-1.1492002
map00071	pt52_A	K07509	-1.747655733
	pt52_A	K07508	-1.1492002
map00230	pt52_A	K00958	1.546657358
	pt52_A	K11808	1.06347646
map00260	pt52_A	K01754	1.17931445
	pt52_A	K00382	1.071539594
map00280	pt52_A	K00382	1.071539594
	pt52_A	K07509	-1.747655733
	pt52_A	K07508	-1.1492002
map00562	pt52_A	K00915	-2.225110517
	pt52_A	K01803	-1.091345651
map00600	pt52_A	K00654	2.159783816
	pt52_A	K04718	1.073444451
map00620	pt52_A	K11262	1.403861653
	pt52_A	K00382	1.071539594
	pt52_A	K01638	-1.296026521
map00630	pt52_A	K00382	1.071539594
	pt52_A	K01638	-1.296026521
map00640	pt52_A	K11262	1.403861653
	pt52_A	K00382	1.071539594
map00710	pt52_A	K00134	2.907928424
	pt52_A	K01803	-1.091345651
map00860	pt52_A	K01749	-4.221923084
	pt52_A	K01698	-2.434236383
	pt52_A	K10960	-1.593846961

	pt52_A	K01845	-1.809267773
	pt52_A	K03428	-2.800053552
	pt52_A	K00231	-1.511170589
	pt52_A	K03403	-1.13437956
	pt52_A	K01885	-1.062977561
map00900	pt52_A	K13789	-2.46751381
	pt52_A	K10960	-1.593846961
map00910	pt52_A	K00262	-1.867669575
	pt52_A	K02575	-1.558038852
map00970	pt52_A	K01881	-1.201217503
	pt52_A	K01885	-1.062977561
map03018	pt52_A	K00962	2.76274854
	pt52_A	K04043	-1.153314853
map03060	pt52_A	K09490	-1.543090472
	pt52_A	K03217	-1.107223113
	pt52_A	K03116	-1.281394436
map03070	pt52_A	K03217	-1.107223113
	pt52_A	K03116	-1.281394436
map04020	pt52_A	K02183	1.240854882
	pt52_A	K04718	1.073444451
map04070	pt52_A	K02183	1.240854882
	pt52_A	K00915	-2.225110517
map04071	pt52_A	K00654	2.159783816
	pt52_A	K04718	1.073444451
map04141	pt52_A	K13993	-2.764062725
	pt52_A	K09487	-2.222194749
	pt52_A	K09490	-1.543090472
	pt52_A	K08057	-1.4336138
	pt52_A	K14001	-1.169919375
map04371	pt52_A	K02183	1.240854882
	pt52_A	K04718	1.073444451
map04612	pt52_A	K09490	-1.543090472
	pt52_A	K08057	-1.4336138

map04626	pt52_A	K02183	1.240854882
	pt52_A	K09487	-2.222194749
map04666	pt52_A	K05768	1.165476554
	pt52_A	K04718	1.073444451
map00010	pt52_B	K00134	1.95160417
	pt52_B	K01835	1.53279059
	pt52_B	K00927	1.544223522
map00061	pt52_B	K00645	2.203152891
	pt52_B	K09458	1.36198263
map00062	pt52_B	K07508	-1.382629161
	pt52_B	K07515	-1.127619491
map00071	pt52_B	K07508	-1.382629161
	pt52_B	K07515	-1.127619491
map00190	pt52_B	K02267	1.108723159
	pt52_B	K03885	-1.122897642
map00280	pt52_B	K07508	-1.382629161
	pt52_B	K07515	-1.127619491
map00520	pt52_B	K01835	1.53279059
	pt52_B	K01809	1.068698574
map00564	pt52_B	K01094	1.466771316
	pt52_B	K00111	1.096129974
map00710	pt52_B	K01595	2.153111441
	pt52_B	K00134	1.95160417
	pt52_B	K00927	1.544223522
	pt52_B	K00855	1.235551302
map00860	pt52_B	K10960	1.714892237
	pt52_B	K03403	1.506462991
	pt52_B	K01845	1.359209815
	pt52_B	K21480	1.621663777
map00910	pt52_B	K02575	1.99558246
	pt52_B	K00262	-1.049605969
map04066	pt52_B	K00134	1.95160417
	pt52_B	K00927	1.544223522

map04142	pt52_B	K12385	-1.521768492
	pt52_B	K12307	-1.422481669
map00010	pt55_3	K00382	-3.423892356
	pt55_3	K00129	-4.899796744
	pt55_3	K00873	-3.004092949
	pt55_3	K00927	-1.963988994
	pt55_3	K01835	-1.425715581
	pt55_3	K01834	-1.287850449
	pt55_3	K01689	-1.220895506
	pt55_3	K01624	-1.001903951
map00020	pt55_3	K00382	-3.423892356
	pt55_3	K01648	-1.395178972
map00030	pt55_3	K00615	-1.703769474
	pt55_3	K01835	-1.425715581
	pt55_3	K01624	-1.001903951
map00052	pt55_3	K01835	-1.425715581
	pt55_3	K12447	-2.0669891
map00053	pt55_3	K12447	-2.0669891
	pt55_3	K00434	-1.216806481
map00061	pt55_3	K11262	-2.151931378
	pt55_3	K15013	-1.539582517
	pt55_3	K00208	-3.534871244
map00195	pt55_3	K02636	-1.120383328
	pt55_3	K02641	-1.634806224
	pt55_3	K02719	-1.287344538
	pt55_3	K08901	-1.158650642
	pt55_3	K08906	-1.143813343
map00220	pt55_3	K00262	1.083912973
	pt55_3	K01948	-1.430346495
	pt55_3	K01755	-1.615614887
	pt55_3	K00620	-1.260153833
map00230	pt55_3	K10807	-2.235674462
	pt55_3	K01835	-1.425715581

	pt55_3	K00958	-1.644976159
	pt55_3	K00365	-1.497534408
map00250	pt55_3	K00262	1.083912973
	pt55_3	K01948	-1.430346495
	pt55_3	K01755	-1.615614887
	pt55_3	K01953	-1.667313253
map00260	pt55_3	K00382	-3.423892356
	pt55_3	K01834	-1.287850449
map00480	pt55_3	K10807	-2.235674462
	pt55_3	K01256	-1.145767644
	pt55_3	K00434	-1.216806481
	pt55_3	K00383	-1.073377711
map00520	pt55_3	K01835	-1.425715581
	pt55_3	K12447	-2.0669891
map00620	pt55_3	K00382	-3.423892356
	pt55_3	K01595	-2.818493846
	pt55_3	K00873	-3.004092949
	pt55_3	K11262	-2.151931378
map00640	pt55_3	K00382	-3.423892356
	pt55_3	K11262	-2.151931378
map00680	pt55_3	K01595	-2.818493846
	pt55_3	K01834	-1.287850449
	pt55_3	K01689	-1.220895506
	pt55_3	K01624	-1.001903951
map00710	pt55_3	K01595	-2.818493846
	pt55_3	K00855	-2.063031027
	pt55_3	K00615	-1.703769474
	pt55_3	K00927	-1.963988994
	pt55_3	K01624	-1.001903951
map00790	pt55_3	K06897	1.528301819
	pt55_3	K00357	1.318209787
map00860	pt55_3	K10960	-1.246260506
	pt55_3	K01599	-1.352264297

map00910	pt55_3	K00262	1.083912973
	pt55_3	K00366	-1.568010652
	pt55_3	K01948	-1.430346495
map03018	pt55_3	K01689	-1.220895506
	pt55_3	K00962	-3.689124087
map04066	pt55_3	K00927	-1.963988994
	pt55_3	K01689	-1.220895506
map04141	pt55_3	K13993	3.685492356
	pt55_3	K09487	1.033338937
	pt55_3	K09503	-1.109538764
map04142	pt55_3	K08568	1.685940719
	pt55_3	K12385	-1.519846523
	pt55_3	K12307	-1.163335383
map04146	pt55_3	K04564	-2.525702928
	pt55_3	K00624	-1.928064552
	pt55_3	K13348	-1.102113105
map04613	pt55_3	K11253	-3.746163384
	pt55_3	K11252	-4.057905995
	pt55_3	K05863	-1.108104866
map00010	pt55_7	K00134	2.17702796
	pt55_7	K00927	1.80996047
	pt55_7	K00895	1.123969731
map00020	pt55_7	K00234	1.290913398
	pt55_7	K00235	1.160458462
	pt55_7	K01958	-1.203027731
	pt55_7	K01648	-1.175381999
map00051	pt55_7	K00895	1.123969731
	pt55_7	K05305	-1.523217923
map00190	pt55_7	K00234	1.290913398
	pt55_7	K00235	1.160458462
	pt55_7	K03949	1.156412163
map00195	pt55_7	K02719	1.598042502
	pt55_7	K02636	1.108645642

map00710	pt55_7	K00134	2.17702796
	pt55_7	K00927	1.80996047
map00860	pt55_7	K00218	1.709237318
	pt55_7	K03403	1.299599153
	pt55_7	K01845	1.402646384
	pt55_7	K04040	1.355587161
	pt55_7	K01599	1.691823906
	pt55_7	K01698	1.674157057
	pt55_7	K01749	1.35977148
map00900	pt55_7	K13789	1.953918751
	pt55_7	K01662	1.075286901
map03030	pt55_7	K02540	-2.154303498
	pt55_7	K02320	-1.076446547
map03050	pt55_7	K03063	1.251425489
	pt55_7	K02728	1.333314819
map04066	pt55_7	K00134	2.17702796
	pt55_7	K00927	1.80996047
map04110	pt55_7	K02540	-2.154303498
	pt55_7	K02604	-1.400923966
map04111	pt55_7	K02540	-2.154303498
	pt55_7	K06677	-1.304965109
	pt55_7	K06674	-1.118915969
	pt55_7	K02604	-1.400923966
map04141	pt55_7	K13993	2.73480405
	pt55_7	K08057	1.458293394
	pt55_7	K09490	1.246532857
	pt55_7	K09503	1.20163307
map04612	pt55_7	K08057	1.458293394
	pt55_7	K09490	1.246532857
map04714	pt55_7	K00234	1.290913398
	pt55_7	K00235	1.160458462
	pt55_7	K03949	1.156412163

Table 8 Frequency of different modules

ModuleColor	Frequency
black	2099
blue	1367
cyan	1430
green	1006
greenyellow	587
grey	11
magenta	742
midnightblue	429
pink	759
purple	2122
salmon	483
tan	535
yellow	1232

Table 9 Phenotypic core genes

Phenotype	Pathway	Gene ID	KEGG ID
FA.18bi3n3.	Protein export	PHATRDRAFT_43657	K03217
	Protein processing in endoplasmic reticulum	PHATRDRAFT_16786	K09487
	Inositol phosphate metabolism	PHATRDRAFT_44782	K00915
	Metabolic pathways	PHATRDRAFT_44782	K00915
	Phosphatidylinositol signaling system	PHATRDRAFT_44782	K00915
	Ubiquitin mediated proteolysis	PHATRDRAFT_54460	K03178
FA.20bi4n6c.	Protein export	PHATRDRAFT_43657	K03217
	Protein processing in endoplasmic reticulum	PHATRDRAFT_16786	K09487
	Inositol phosphate metabolism	PHATRDRAFT_44782	K00915
	Metabolic pathways	PHATRDRAFT_44782	K00915
	Phosphatidylinositol signaling system	PHATRDRAFT_44782	K00915
	Ubiquitin mediated proteolysis	PHATRDRAFT_54460	K03178
FA.22bi1n9c	Inositol phosphate metabolism	PHATRDRAFT_44782	K00915
	Metabolic pathways	PHATRDRAFT_44782	K00915

	Phosphatidylinositol signaling system	PHATRDRAFT_44782	K00915
FA.22bi5n3or24bi1n9c.	Protein export	PHATRDRAFT_43657	K03217
	Protein processing in endoplasmic reticulum	PHATRDRAFT_16786	K09487
	Inositol phosphate metabolism	PHATRDRAFT_44782	K00915
	Metabolic pathways	PHATRDRAFT_44782	K00915
	Phosphatidylinositol signaling system	PHATRDRAFT_44782	K00915
	Ubiquitin mediated proteolysis	PHATRDRAFT_54460	K03178
FA.3OHC14.	Sphingolipid metabolism	PHATRDRAFT_44806	K04718
	Metabolic pathways	PHATRDRAFT_44806	K04718
FA.C20.	Sphingolipid metabolism	PHATRDRAFT_44806	K04718
	Metabolic pathways	PHATRDRAFT_44806	K04718
		PHATRDRAFT_45509	K01950
	Nicotinate and nicotinamide metabolism	PHATRDRAFT_45509	K01950
	Biosynthesis of cofactors	PHATRDRAFT_45509	K01950
FA.Totals.	Sphingolipid metabolism	PHATRDRAFT_44806	K04718
	Metabolic pathways	PHATRDRAFT_44806	K04718
		PHATRDRAFT_45509	K01950
	Nicotinate and nicotinamide metabolism	PHATRDRAFT_45509	K01950
	Biosynthesis of cofactors	PHATRDRAFT_45509	K01950
Mu.cells.day.Fusiform	Oxidative phosphorylation	PHATRDRAFT_bd1704	K03950
	Metabolic pathways	PHATRDRAFT_bd1704	K03950
Lambda.days.Fusiform	Glycolysis / Gluconeogenesis	PHATRDRAFT_26201	K01834
	Glycine, serine and threonine metabolism	PHATRDRAFT_26201	K01834
	Metabolic pathways	PHATRDRAFT_26201	K01834
		PHATRDRAFT_bd1367	K13800
		PHATRDRAFT_bd1704	K03950
	Biosynthesis of secondary metabolites	PHATRDRAFT_26201	K01834
	Carbon metabolism	PHATRDRAFT_26201	K01834
	Biosynthesis of amino acids	PHATRDRAFT_26201	K01834
	Pyrimidine metabolism	PHATRDRAFT_bd1367	K13800
	Nucleotide metabolism	PHATRDRAFT_bd1367	K13800
	Biosynthesis of cofactors	PHATRDRAFT_bd1367	K13800
	Oxidative phosphorylation	PHATRDRAFT_bd1704	K03950

A.cells.Oval	Metabolic pathways	PHATRDRAFT_bd1704	K03950
Mu.cells.day.Total	Pyrimidine metabolism	PHATRDRAFT_bd1367	K13800
	Metabolic pathways	PHATRDRAFT_bd1367	K13800
		PHATRDRAFT_bd1704	K03950
	Nucleotide metabolism	PHATRDRAFT_bd1367	K13800
	Biosynthesis of cofactors	PHATRDRAFT_bd1367	K13800
	Oxidative phosphorylation	PHATRDRAFT_bd1704	K03950
A.cells.Total	Glycolysis / Gluconeogenesis	PHATRDRAFT_48983	K00927
		PHATRDRAFT_22122	K00134
	Carbon fixation in photosynthetic organisms	PHATRDRAFT_48983	K00927
		PHATRDRAFT_22122	K00134
	Metabolic pathways	PHATRDRAFT_48983	K00927
		PHATRDRAFT_5902	K21480
		PHATRDRAFT_22122	K00134
	Biosynthesis of secondary metabolites	PHATRDRAFT_48983	K00927
		PHATRDRAFT_5902	K21480
		PHATRDRAFT_22122	K00134
	Carbon metabolism	PHATRDRAFT_48983	K00927
		PHATRDRAFT_22122	K00134
	Biosynthesis of amino acids	PHATRDRAFT_48983	K00927
		PHATRDRAFT_22122	K00134
	Porphyrin metabolism	PHATRDRAFT_5902	K21480
	ABC transporters	PHATRDRAFT_21548	K05658
	Calcium signaling pathway	PHATRDRAFT_J22873	K05863
FA.C14.	Oxidative phosphorylation	PHATRDRAFT_18398	K02136
	Metabolic pathways	PHATRDRAFT_18398	K02136
		PHATRDRAFT_13951	K00262
	Ribosome biogenesis in eukaryotes	PHATRDRAFT_2182	K14538
	Folate biosynthesis	PHATRDRAFT_44454	K06897
	Biosynthesis of cofactors	PHATRDRAFT_44454	K06897
	Arginine biosynthesis	PHATRDRAFT_13951	K00262
	Alanine, aspartate and glutamate metabolism	PHATRDRAFT_13951	K00262
	Nitrogen metabolism	PHATRDRAFT_13951	K00262

FA.16bi1.	Folate biosynthesis	PHATRDRAFT_44454	K06897
	Biosynthesis of cofactors	PHATRDRAFT_44454	K06897
	Arginine biosynthesis	PHATRDRAFT_13951	K00262
	Alanine, aspartate and glutamate metabolism	PHATRDRAFT_13951	K00262
	Nitrogen metabolism	PHATRDRAFT_13951	K00262
	Metabolic pathways	PHATRDRAFT_13951	K00262
FA.17bi1or3OHC12.	Ribosome biogenesis in eukaryotes	PHATRDRAFT_2182	K14538
	Arginine biosynthesis	PHATRDRAFT_13951	K00262
	Alanine, aspartate and glutamate metabolism	PHATRDRAFT_13951	K00262
	Nitrogen metabolism	PHATRDRAFT_13951	K00262
	Metabolic pathways	PHATRDRAFT_13951	K00262
FA.2OHC12.	Photosynthesis	PHATRDRAFT_54499	K08901
	Metabolic pathways	PHATRDRAFT_54499	K08901
FA.16bi2n4.	Metabolic pathways	PHATRDRAFT_22956	K08907
		PHATRDRAFT_49167	K00383
		PHATRDRAFT_26293	K02719
		PHATRDRAFT_44601	K08910
		PHATRDRAFT_54499	K08901
		PHATRDRAFT_20331	K02716
		PHATRDRAFT_12762	K00620
	Glutathione metabolism	PHATRDRAFT_49167	K00383
	Photosynthesis	PHATRDRAFT_26293	K02719
		PHATRDRAFT_54499	K08901
		PHATRDRAFT_20331	K02716
	Arginine biosynthesis	PHATRDRAFT_12762	K00620
	Biosynthesis of secondary metabolites	PHATRDRAFT_12762	K00620
	2-Oxocarboxylic acid metabolism	PHATRDRAFT_12762	K00620
	Biosynthesis of amino acids	PHATRDRAFT_12762	K00620
FA.18bi1n9c.	Metabolic pathways	PHATRDRAFT_22956	K08907
FA.18bi2n6c.	Photosynthesis	PHATRDRAFT_54499	K08901
	Metabolic pathways	PHATRDRAFT_54499	K08901
FA.22bi2n6c.	Metabolic pathways	PHATRDRAFT_22956	K08907
		PHATRDRAFT_49167	K00383

		PHATRDRAFT_26293	K02719
		PHATRDRAFT_44601	K08910
		PHATRDRAFT_54499	K08901
		PHATRDRAFT_20331	K02716
	Glutathione metabolism	PHATRDRAFT_49167	K00383
	Photosynthesis	PHATRDRAFT_26293	K02719
		PHATRDRAFT_54499	K08901
		PHATRDRAFT_20331	K02716
FA.22bi6n3c.	Photosynthesis	PHATRDRAFT_26293	K02719
		PHATRDRAFT_54499	K08901
		PHATRDRAFT_20331	K02716
	Metabolic pathways	PHATRDRAFT_26293	K02719
		PHATRDRAFT_54499	K08901
		PHATRDRAFT_20331	K02716
A.cells.Triradiate	DNA replication	PHATRDRAFT_bd1073	K02320
Mu.cells.day.Triradiate	DNA replication	PHATRDRAFT_bd1073	K02320
Lambda.days.Triradiate	DNA replication	PHATRDRAFT_bd1073	K02320
FA.C8.	DNA replication	PHATRDRAFT_bd1073	K02320
FA.C10.	DNA replication	PHATRDRAFT_bd1073	K02320
FA.C13.	DNA replication	PHATRDRAFT_bd1073	K02320
FA.2OHC10.	DNA replication	PHATRDRAFT_bd1073	K02320
FA.2OHC14.	Oxidative phosphorylation	PHATRDRAFT_49313	K03949
	Metabolic pathways	PHATRDRAFT_49313	K03949
		PHATRDRAFT_32747	K00134
		PHATRDRAFT_32823	K01934
		PHATRDRAFT_51092	K01915
		PHATRDRAFT_29157	K00927
		PHATR_10454	K01074
		PHATR_13358	K02636
		PHATRDRAFT_bd348	K00053
	Glycolysis / Gluconeogenesis	PHATRDRAFT_32747	K00134
		PHATRDRAFT_29157	K00927
	Carbon fixation in photosynthetic organisms	PHATRDRAFT_32747	K00134

	PHATRDRAFT_29157	K00927
Biosynthesis of secondary metabolites	PHATRDRAFT_32747	K00134
	PHATRDRAFT_29157	K00927
	PHATRDRAFT_bd348	K00053
Carbon metabolism	PHATRDRAFT_32747	K00134
	PHATRDRAFT_29157	K00927
Biosynthesis of amino acids	PHATRDRAFT_32747	K00134
	PHATRDRAFT_51092	K01915
	PHATRDRAFT_29157	K00927
	PHATRDRAFT_bd348	K00053
One carbon pool by folate	PHATRDRAFT_32823	K01934
Protein processing in endoplasmic reticulum	PHATRDRAFT_41172	K08057
	PHATRDRAFT_38015	K09503
Phagosome	PHATRDRAFT_41172	K08057
Proteasome	PHATRDRAFT_11735	K03063
Nucleocytoplasmic transport	PHATRDRAFT_28737	K03231
Arginine biosynthesis	PHATRDRAFT_51092	K01915
Alanine, aspartate and glutamate metabolism	PHATRDRAFT_51092	K01915
Glyoxylate and dicarboxylate metabolism	PHATRDRAFT_51092	K01915
Nitrogen metabolism	PHATRDRAFT_51092	K01915
Fatty acid elongation	PHATR_10454	K01074
Fatty acid metabolism	PHATR_10454	K01074
Photosynthesis	PHATR_13358	K02636
Valine, leucine and isoleucine biosynthesis	PHATRDRAFT_bd348	K00053
Pantothenate and CoA biosynthesis	PHATRDRAFT_bd348	K00053
2-Oxocarboxylic acid metabolism	PHATRDRAFT_bd348	K00053

2 Supplementary Figures

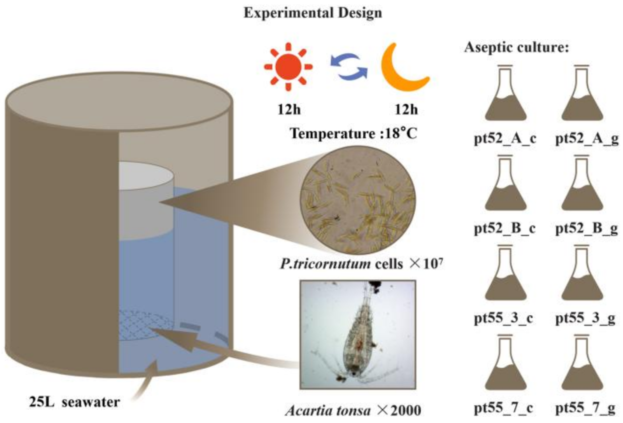

Fig.1 Experimental design concept diagram

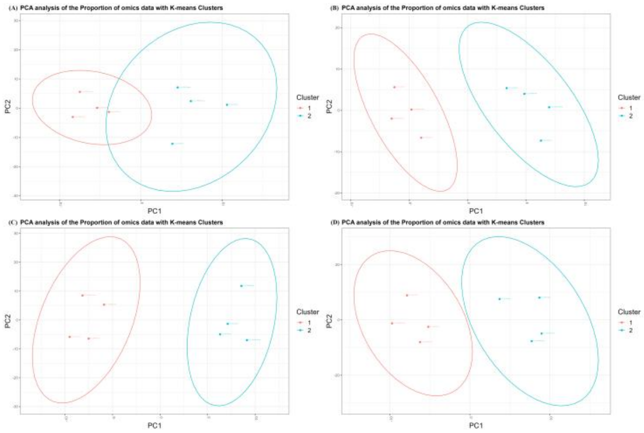

Fig.2 PCA analysis of the Proportion of omics data with K-means Clusters. The dots in the figure show the data clustering of 8 sample groups at four levels (mRNA, LncRNA, Protein, Metabolites). K-means cluster method is used, and K value is set at 2. The name of the sample group is marked next to each dot. The principal components are selected as PC1 and PC2, and the distance between each dot is the clustering case. Subfigure (A) corresponds to pt52_A. Subfigure (B) denotes pt52_B. Subfigure (C) represents pt55_3. Subfigure (D) highlights pt55_7.

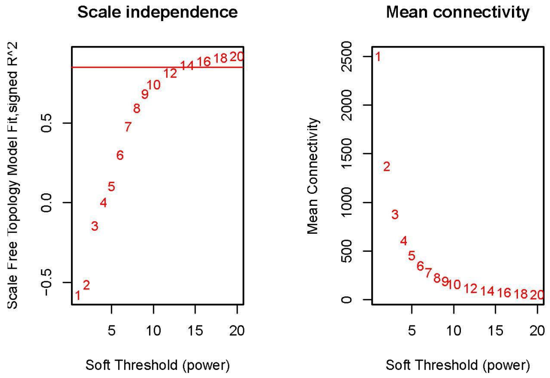

Fig.8 Determination of soft threshold β. The figure on the left depicts the variation of the scale-free topological fitting coefficient R^2 (Y-axis) with respect to the soft threshold β (X-axis). The scale-free topological fitting coefficient corresponding to the red line of sight R^2 is 0.8, and the larger the R^2, the more consistent the scale-free distribution of the weighted gene coexpression network is. The final soft threshold is the β value that makes the scale-free topological fitting coefficient R^2 reach 0.8 for the first time. The figure on the right depicts the mean connectivity (Y-axis) within the coexpression network with respect to the soft threshold β (X-axis). The more the constructed coexpression network conforms to the scale-free distribution law, the closer the average connectivity within the network is to 0. It can be analyzed from the figure that 14 is chosen as the soft threshold in this experiment.

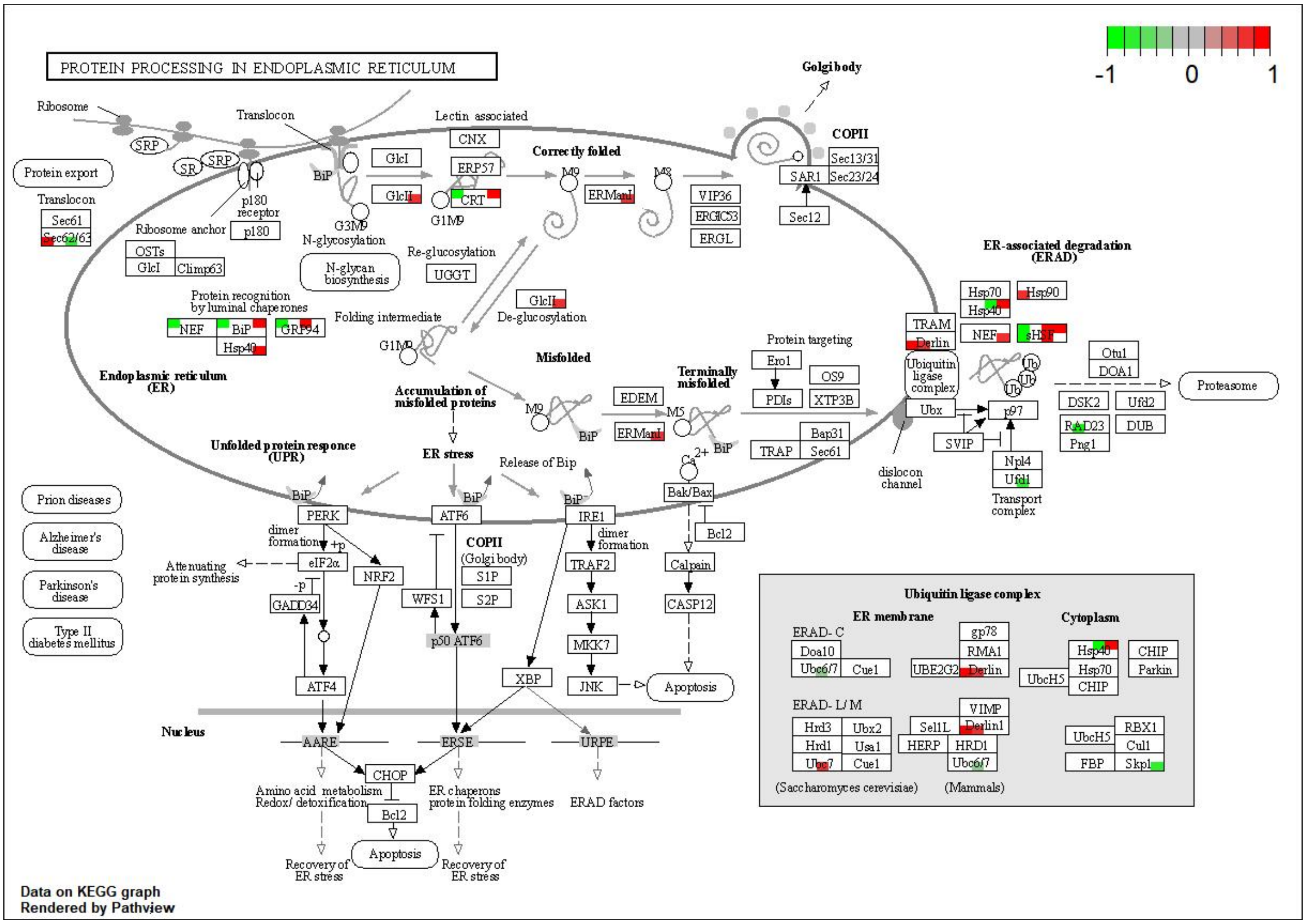

Fig.14 Regulation of Protein Processing in Endoplasmic Reticulum under gazing stress at both transcriptomic and proteomic levels. Each rectangle represents an enzyme or protein, which represents the expression of a gene. The rectangle was evenly divided into 8 parts, with the top four cells representing transcriptomic level expression and the bottom four cells representing proteomic level expression. The rectangle is divided from left to right into four parts, representing the strains: pt52_A, pt52_B, pt55_3, pt55_7. From red to green, red means up-regulation, green means down-regulation, and white means no data.

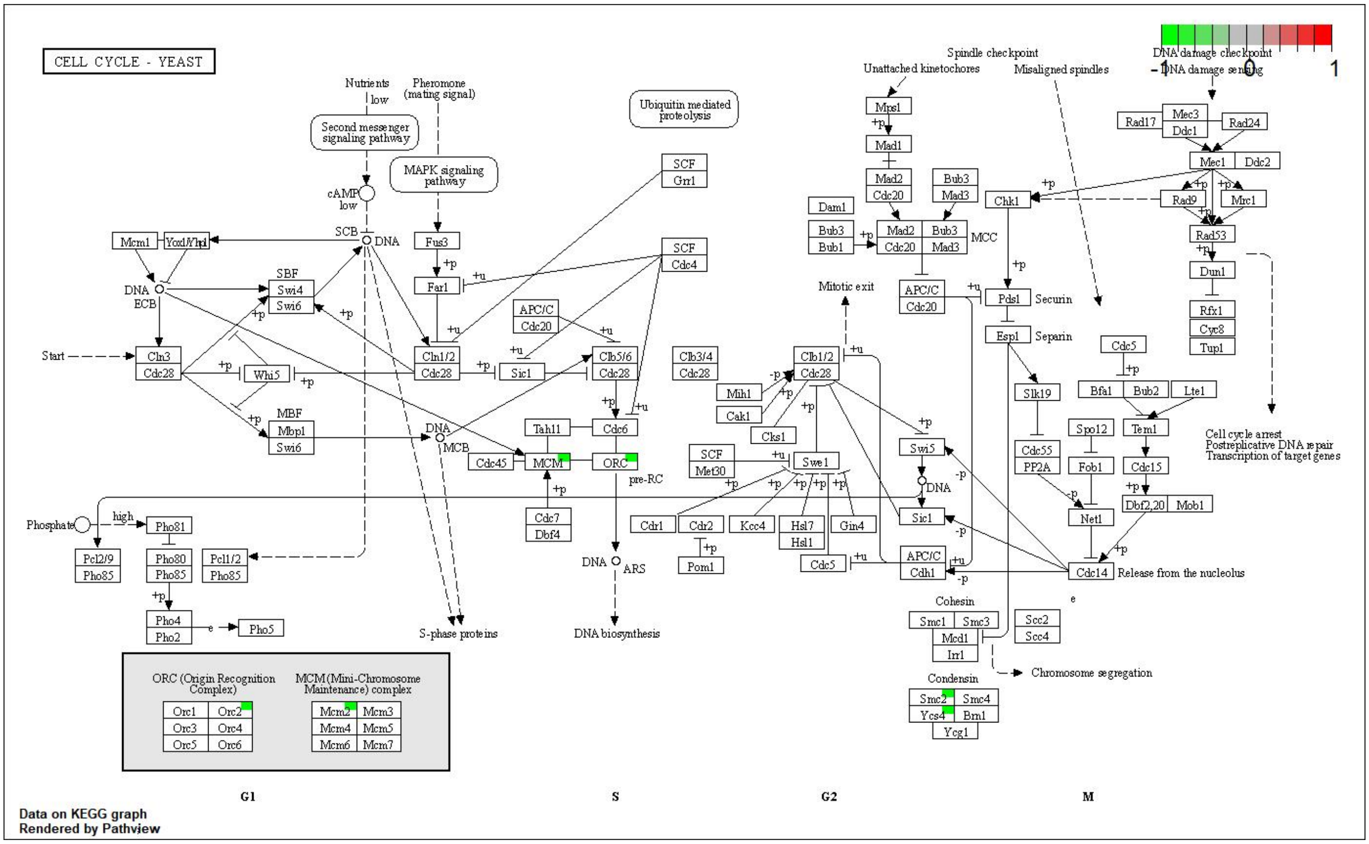

Fig.15 Regulation of Cell Cycle - Yeast under gazing stress at both transcriptomic and proteomic levels. Each rectangle represents an enzyme or protein, which represents the expression of a gene. The rectangle was evenly divided into 8 parts, with the top four cells representing transcriptomic level expression and the bottom four cells representing proteomic level expression. The rectangle is divided from left to right into four parts, representing the strains: pt52_A, pt52_B, pt55_3, pt55_7. From red to green, red means up-regulation, green means down-regulation, and white means no data.

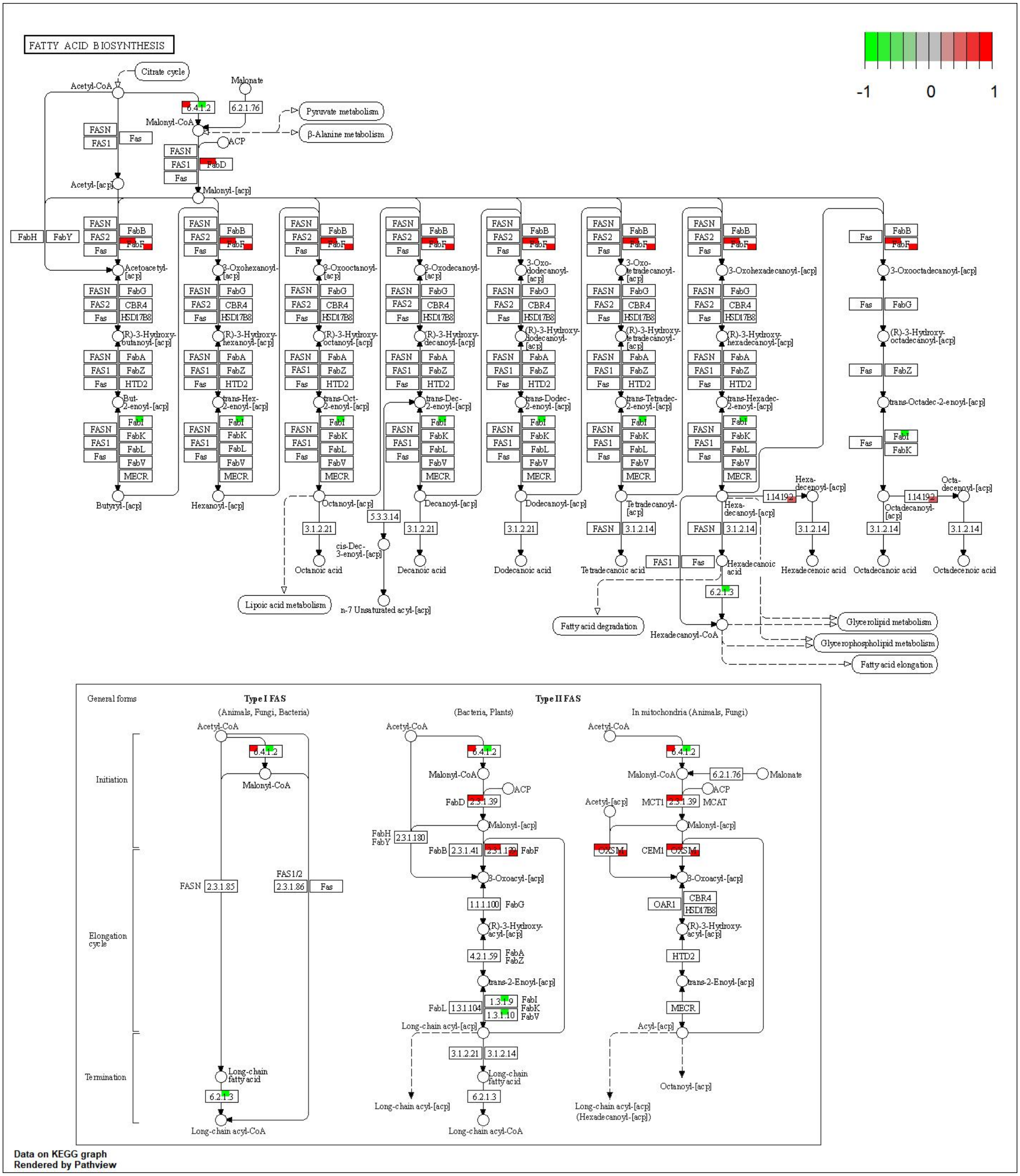

Fig.16 Regulation of Fatty Acid Biosynthesis under gazing stress at both transcriptomic and proteomic levels. Each rectangle represents an enzyme or protein, which represents the expression of a gene. The rectangle was evenly divided into 8 parts, with the top four cells representing transcriptomic level expression and the bottom four cells representing proteomic level expression. The rectangle is divided from left to right into four parts, representing

the strains: pt52_A, pt52_B, pt55_3, pt55_7. From red to green, red means up-regulation, green means down-regulation, and white means no data.

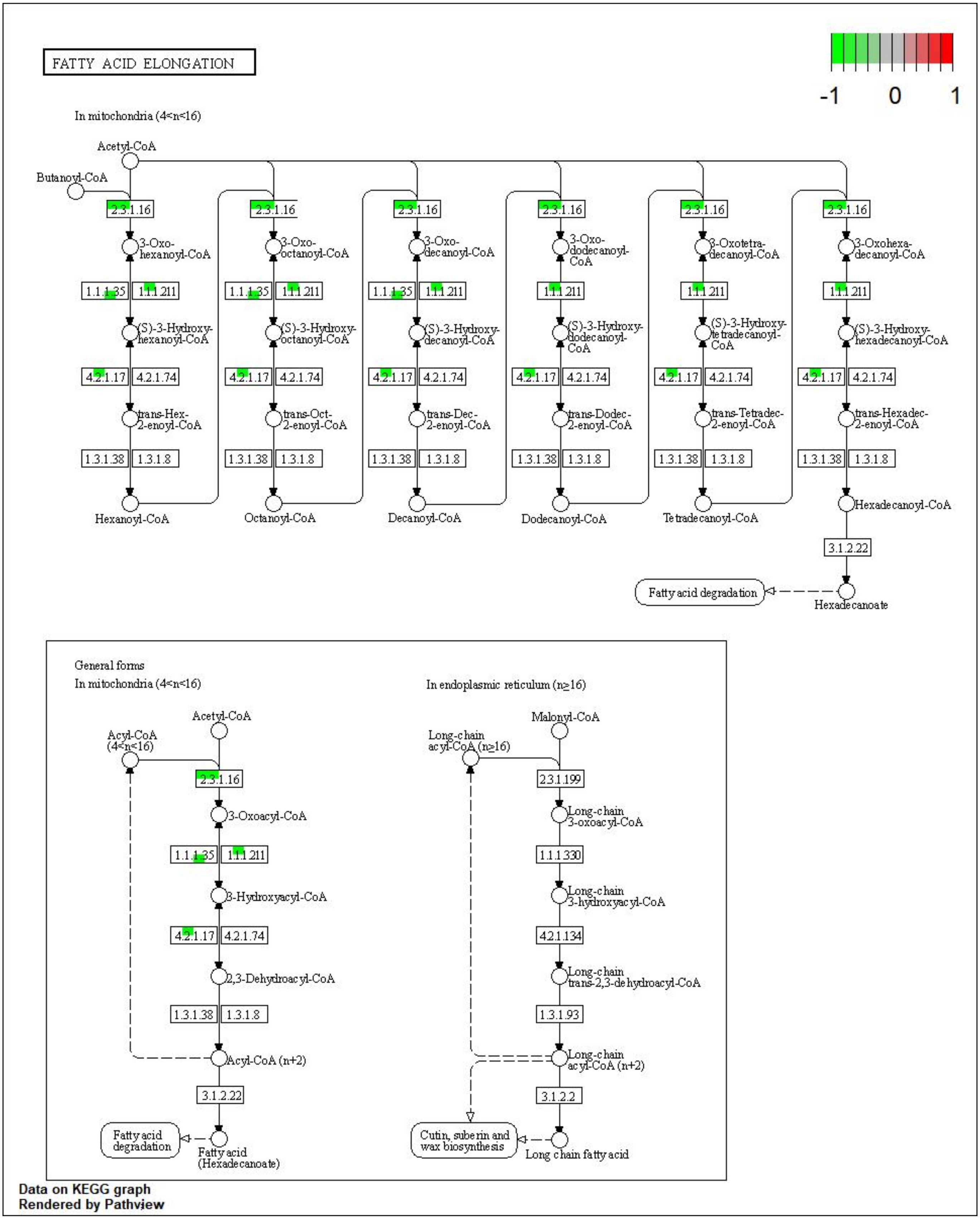
FATTY ACID ELONGATION
-1
0
1
In mitochondria (4<n<16)
Acetyl-CoA
Butanoyl-CoA
2.3.1.16
3-Oxo-hexanoyl-CoA
3-Oxo-octanoyl-CoA
3-Oxo-decanoyl-CoA
3-Oxo-dodecanoyl-CoA
3-Oxotetra-decanoyl-CoA
3-Oxohexa-decanoyl-CoA
1.1.1.35
1.1.1.211
(S)-3-Hydroxy-hexanoyl-CoA
(S)-3-Hydroxy-octanoyl-CoA
(S)-3-Hydroxy-decanoyl-CoA
(S)-3-Hydroxy-dodecanoyl-CoA
(S)-3-Hydroxy-tetradecanoyl-CoA
(S)-3-Hydroxy-hexadecanoyl-CoA
4.2.1.17
4.2.1.74
trans-Hex-2-enoyl-CoA
trans-Oct-2-enoyl-CoA
trans-Dec-2-enoyl-CoA
trans-Dodec-2-enoyl-CoA
trans-Tetradec-2-enoyl-CoA
trans-Hexadec-2-enoyl-CoA
1.3.1.38
1.3.1.8
Hexanoyl-CoA
Octanoyl-CoA
Decanoyl-CoA
Dodecanoyl-CoA
Tetradecanoyl-CoA
Hexadecanoyl-CoA
3.1.2.22
Fatty acid degradation
Hexadecanoate
General forms
In mitochondria (4<n<16)
Acetyl-CoA
Acyl-CoA (4<n<16)
2.3.1.16
3-Oxoacyl-CoA
1.1.1.35
1.1.1.211
3-Hydroxyacyl-CoA
4.2.1.17
4.2.1.74
2,3-Dehydroacyl-CoA
1.3.1.38
1.3.1.8
Acyl-CoA (n+2)
3.1.2.22
Fatty acid degradation
Fatty acid (Hexadecanoate)
In endoplasmic reticulum (n≥16)
Malonyl-CoA
Long-chain acyl-CoA (n≥16)
2.3.1.199
Long-chain 3-oxoacyl-CoA
1.1.1.330
Long-chain 3-hydroxyacyl-CoA
4.2.1.134
Long-chain trans-2,3-dehydroacyl-CoA
1.3.1.93
Long-chain acyl-CoA (n+2)
3.1.2.2
Cutin, suberin and wax biosynthesis
Long chain fatty acid
Data on KEGG graph
Rendered by Pathview

Fig.17 Regulation of Fatty Acid Elongation under gazing stress at both transcriptomic and proteomic levels. Each rectangle represents an enzyme or protein, which represents the expression of a gene. The rectangle was evenly divided into 8 parts, with the top four cells representing transcriptomic level expression and the bottom four cells representing proteomic level expression. The rectangle is divided from left to right into four parts, representing the strains: pt52_A, pt52_B, pt55_3, pt55_7. From red to green, red means up-regulation, green means down-regulation, and white means no data.

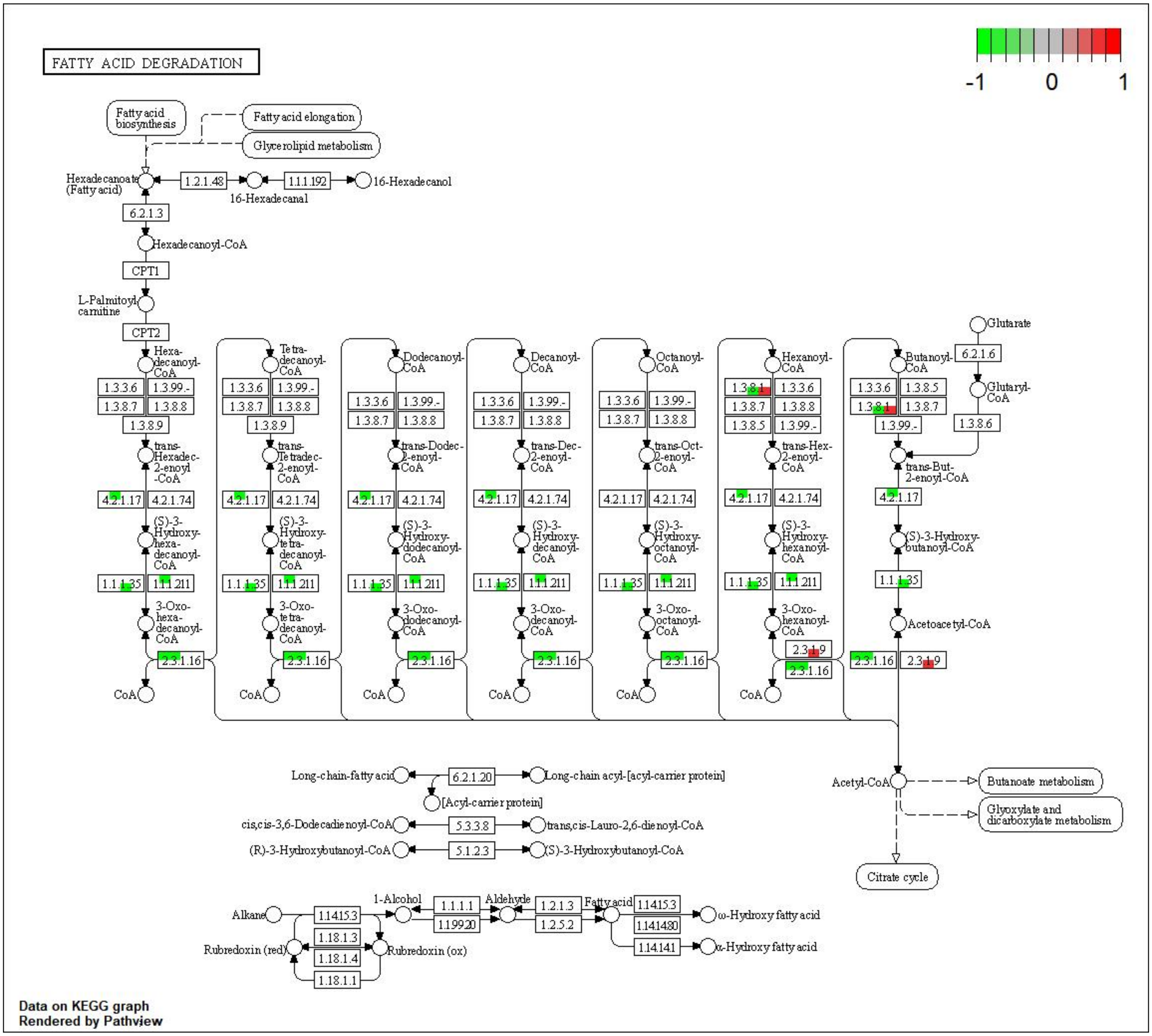

Fig.18 Regulation of Fatty Acid Degradation under gazing stress at both transcriptomic and proteomic levels. Each rectangle represents an enzyme or protein, which represents the expression of a gene. The rectangle was evenly divided into 8 parts, with the top four cells representing transcriptomic level expression and the bottom four cells representing proteomic level expression. The rectangle is divided from left to right into four parts, representing the strains: pt52_A, pt52_B, pt55_3, pt55_7. From red to green, red means up-regulation, green means down-regulation, and white means no data.

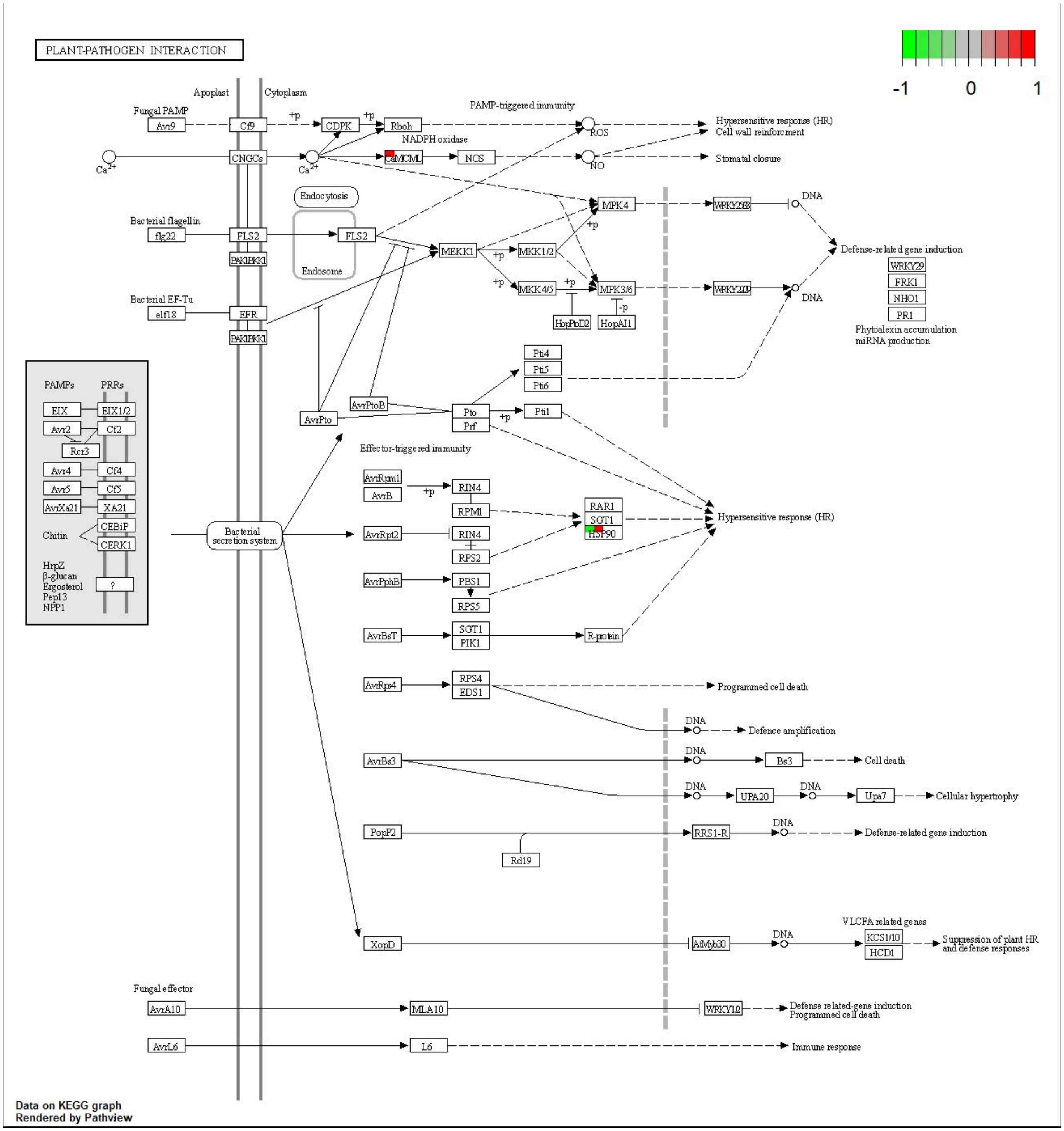

Fig.19 Regulation of Plant-Pathogen Interaction under gazing stress at both transcriptomic and proteomic levels. Each rectangle represents an enzyme or protein, which represents the expression of a gene. The rectangle was evenly divided into 8 parts, with the top four cells representing transcriptomic level expression and the bottom four cells representing proteomic level expression. The rectangle is divided from left to right into four parts, representing the strains: pt52_A, pt52_B, pt55_3, pt55_7. From red to green, red means up-regulation, green means down-regulation, and white means no data.

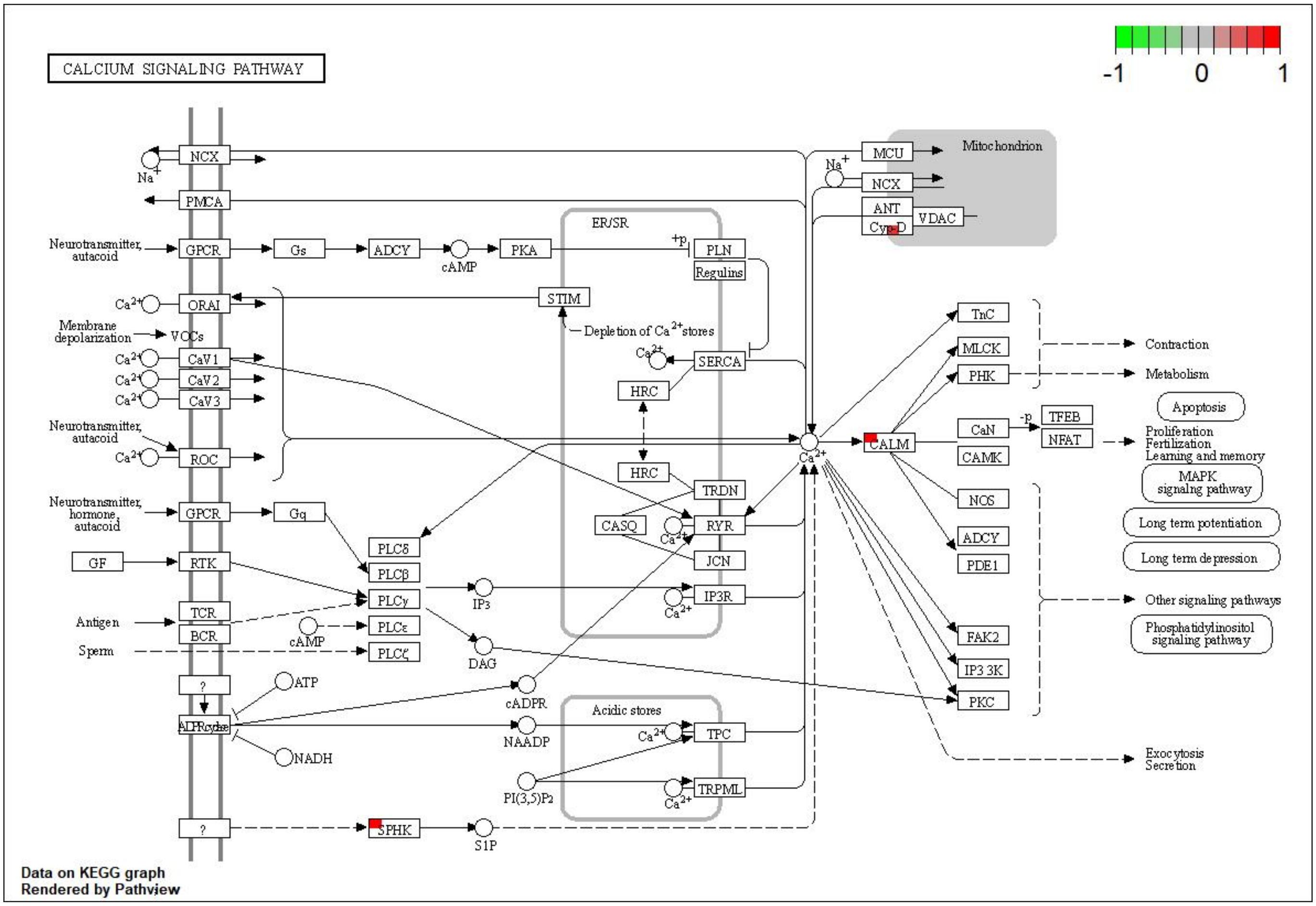

Fig.20 Regulation of Calcium Signaling Pathway under gazing stress at both transcriptomic and proteomic levels. Each rectangle represents an enzyme or protein, which represents the expression of a gene. The rectangle was evenly divided into 8 parts, with the top four cells representing transcriptomic level expression and the bottom four cells representing proteomic level expression. The rectangle is divided from left to right into four parts, representing the strains: pt52_A, pt52_B, pt55_3, pt55_7. From red to green, red means up-regulation, green means down-regulation, and white means no data.

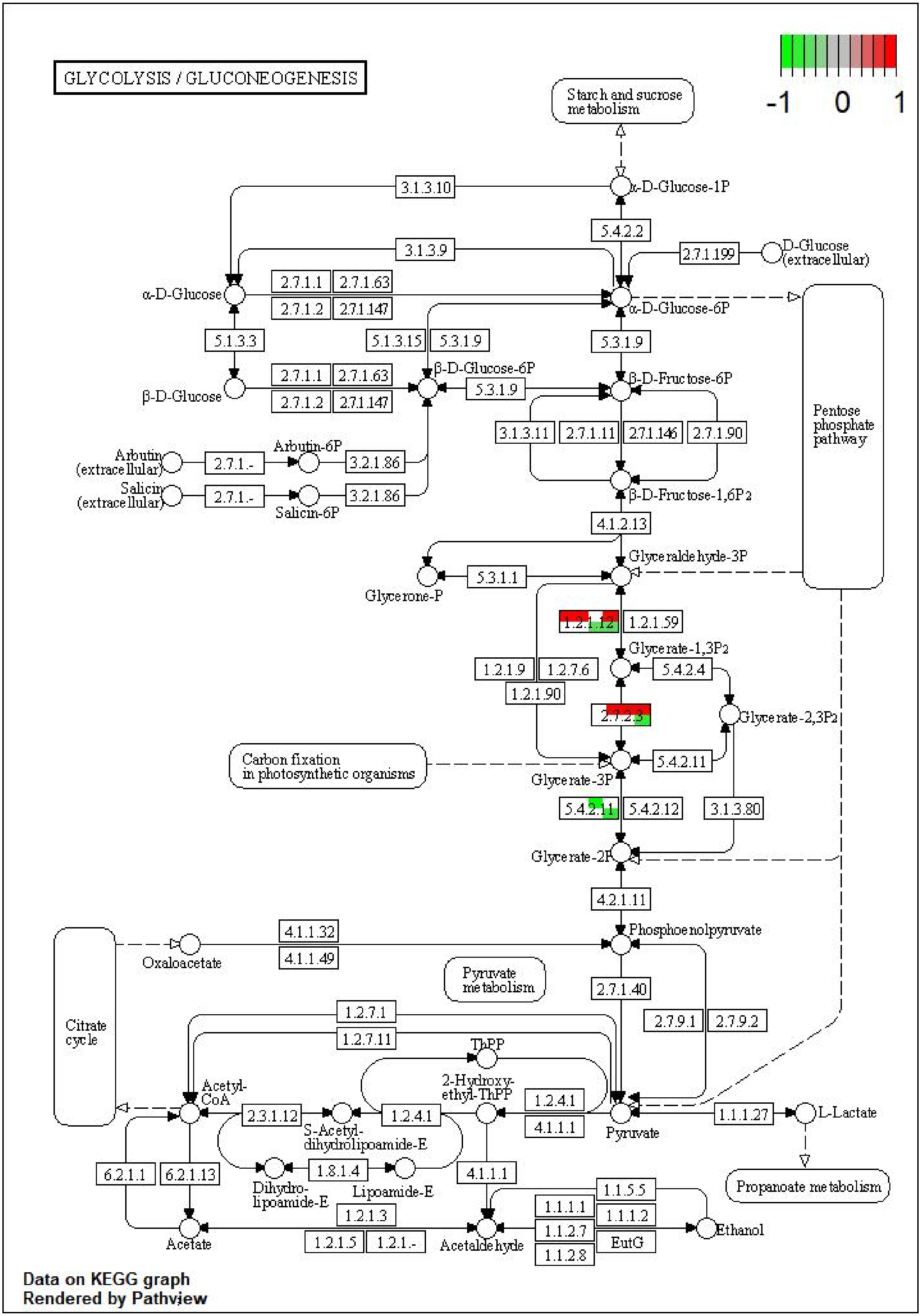
GLYCOLYSIS / GLUCONEOGENESIS
-1 0 1
Starch and sucrose metabolism
α-D-Glucose-1P
3.1.3.10
5.4.2.2
3.1.3.9
2.7.1.199
D-Glucose (extracellular)
α-D-Glucose
2.7.1.1
2.7.1.63
2.7.1.2
2.7.1.147
α-D-Glucose-6P
5.1.3.3
5.1.3.15
5.3.1.9
β-D-Glucose-6P
β-D-Fructose-6P
β-D-Glucose
3.1.3.11
2.7.1.11
2.7.1.146
2.7.1.90
Pentose phosphate pathway
Arbutin (extracellular)
2.7.1.-
Arbutin-6P
3.2.1.86
Salicin (extracellular)
Salicin-6P
β-D-Fructose-1,6P2
4.1.2.13
Glyceraldehyde-3P
5.3.1.1
Glycerone-P
1.2.1.12
1.2.1.59
Glycerate-1,3P2
1.2.1.9
1.2.7.6
5.4.2.4
1.2.1.90
2.7.2.3
Glycerate-2,3P2
Carbon fixation in photosynthetic organisms
5.4.2.11
Glycerate-3P
5.4.2.12
3.1.3.80
Glycerate-2P
4.2.1.11
Phosphoenolpyruvate
4.1.1.32
4.1.1.49
Oxaloacetate
Citrate cycle
Pyruvate metabolism
2.7.1.40
2.7.9.1
2.7.9.2
1.2.7.1
1.2.7.11
ThPP
Acetyl-CoA
2-Hydroxy-ethyl-ThPP
1.2.4.1
2.3.1.12
S-Acetyl-dihydrolipoamide-E
4.1.1.1
Pyruvate
1.1.1.27
L-Lactate
6.2.1.1
6.2.1.13
1.8.1.4
Dihydro-lipoamide-E
Lipoamide-E
1.1.5.5
Propanoate metabolism
1.1.1.1
1.1.1.2
1.2.1.3
1.1.2.7
Ethanol
EutG
1.1.2.8
Acetate
1.2.1.5
1.2.1.-
Acetaldehyde
Data on KEGG graph
Rendered by Pathview

Fig.21 Regulation of Glycolysis/Gluconeogenesis under gazing stress at both transcriptomic and proteomic levels. Each rectangle represents an enzyme or protein, which represents the expression of a gene. The rectangle was evenly divided into 8 parts, with the top four cells representing transcriptomic level expression and the bottom four cells representing proteomic level expression. The rectangle is divided from left to right into four parts, representing the strains: pt52_A, pt52_B, pt55_3, pt55_7. From red to green, red means up-regulation, green means down-regulation, and white means no data.

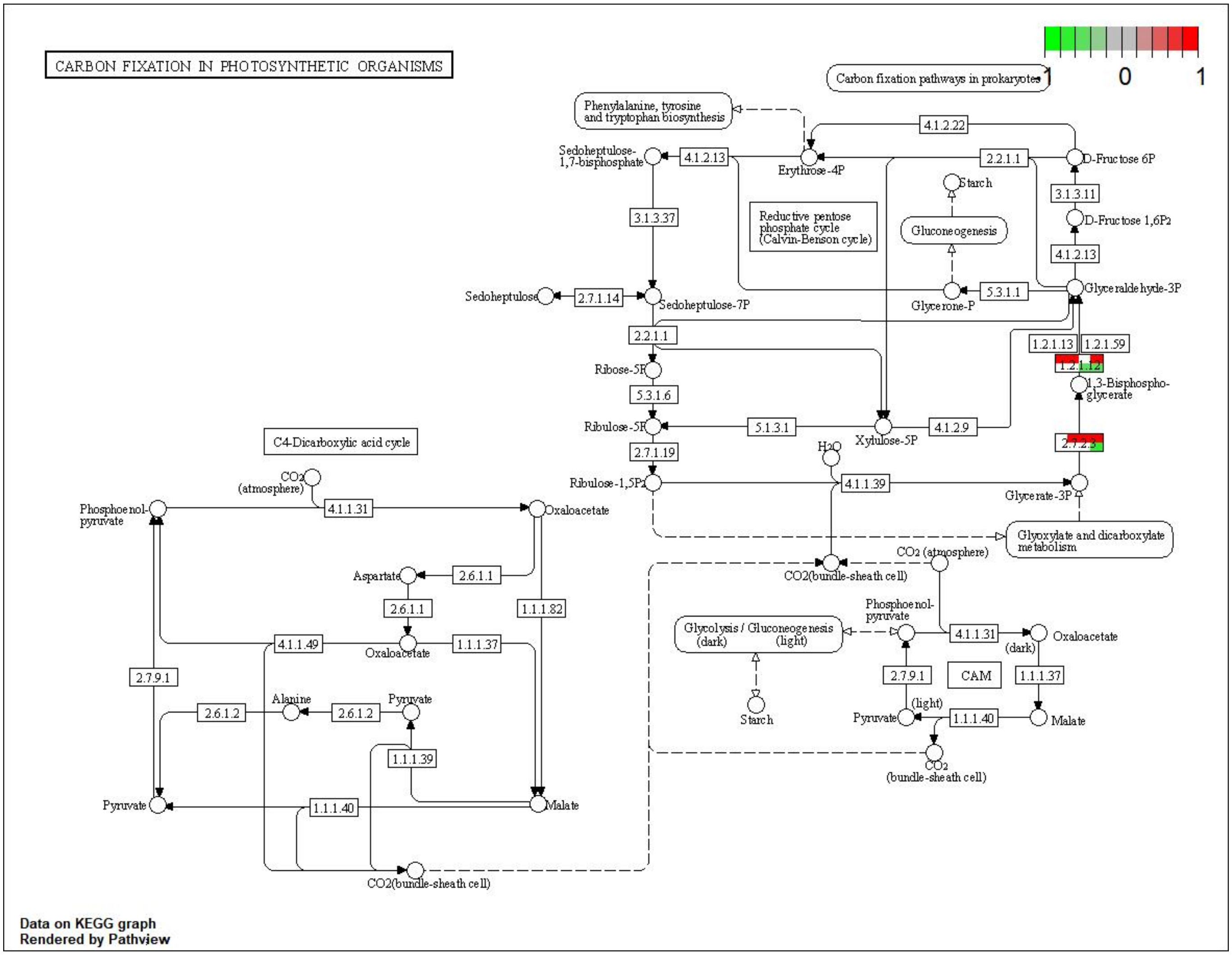

Fig.22 Regulation of Carbon Fixation in Photosynthetic Organisms under gazing stress at both transcriptomic and proteomic levels. Each rectangle represents an enzyme or protein, which represents the expression of a gene. The rectangle was evenly divided into 8 parts, with the top four cells representing transcriptomic level expression and the bottom four cells representing proteomic level expression. The rectangle is divided from left to right into four parts, representing the strains: pt52_A, pt52_B, pt55_3, pt55_7. From red to green, red means up-regulation, green means down-regulation, and white means no data.

3 R Codes

3.1 R codes of WGCNA

3.1.1 Soft Threshold

```
plot(x=sft$fitIndices[,1],
y=-sign(sft$fitIndices[,3])*sft$fitIndices[,2],
xlab="Soft Threshold (power)",
ylab="Scale Free Topology Model Fit, signed R^2",type="n",
main = paste("Scale independence"))
text(sft$fitIndices[,1], -sign(sft$fitIndices[,3])*sft$fitIndices[,2],
labels=powers,cex=0.9,col="red")
abline(h=0.80,col="red")
```

3.1.2 Module And Traits

```
library(WGCNA, warn.conflicts = F);
library(hash);
networktype = "signed hybrid";
softPower = 14;
getwd();
workingdir = "   ";
setwd(workingdir);
options(stringsAsFactors = F);
enableWGCNAThreads();
nGenes = ncol(datExpr);
nSamples = nrow(datExpr);
MEs0 = moduleEigengenes(datExpr, moduleColors)$eigengenes;
MEs = orderMEs(MEs0);
tempdf = as.data.frame(datTraits[,1]);
names(tempdf) = names(datTraits)[1];
AllgeneTraitSignificance = as.data.frame(cor(datExpr, tempdf, use = "p"));
for(i in 2:length(datTraits)){
  tempdf = as.data.frame(datTraits[,i]);
  names(tempdf) = names(datTraits)[i];
  geneTraitSignificance = as.data.frame(cor(datExpr, tempdf, use = "p"));
  if(is.na(geneTraitSignificance[1,1])){
```

```
    next;
  }
  AllgeneTraitSignificance = cbind(AllgeneTraitSignificance, geneTraitSignificance);
}
datKME = signedKME(datExpr, MEs, outputColumnName = "MM.");
Hash = hash(keys = names(AllgeneTraitSignificance),
            values = c("MM.blue","MM.purple", "MM.purple", "MM.purple","MM.purple","    ", "    ", "
"));
for( i in names(AllgeneTraitSignificance)){
  if(Hash[[i]] == "MM.black"){
    MM.black = append(MM.black, i);
  }else if(Hash[[i]] == "MM.blue"){
    MM.blue = append(MM.blue, i);
  }else if(Hash[[i]] == "MM.greenyellow"){
    MM.greenyellow = append(MM.greenyellow, i);
  }else if(Hash[[i]] == "MM.midnightblue"){
    MM.midnightblue = append(MM.midnightblue, i);
  }else if(Hash[[i]] == "MM.pink"){
    MM.pink = append(MM.pink, i);
  }else if(Hash[[i]] == "MM.purple"){
    MM.purple = append(MM.purple, i);
  }else if(Hash[[i]] == "MM.salmon"){
    MM.salmon = append(MM.salmon, i);
  }else{
    MM.yellow = append(MM.yellow, i);
  }
}
save(file = "   ",
MM.black, MM.blue, MM.greenyellow, MM.midnightblue,MM.pink, MM.yellow, MM.salmon,
MM.purple);
workingdir = "ModuleHubGene/"
setwd(workingdir);
```

```
for(i in 1:length(AllgeneTraitSignificance)){
  FilterGenes = abs(datKME[,names(datKME) == Hash[[names(AllgeneTraitSignificance)[i]]]]) >
0.8 &
    abs(AllgeneTraitSignificance[,i]) > 0.8;
  hubgene = as.data.frame(dimnames(data.frame(datExpr))[[2]][FilterGenes]);
  names(hubgene) = "geneName";
  write.csv(file = paste(names(AllgeneTraitSignificance)[i],
                         Hash[[names(AllgeneTraitSignificance)[i]]],
                         sep = "_", ".csv"), hubgene);
  print("done");
}
```

3.1.3 Hub Gene Filter by Pvalue

```
library(KEGGREST);
library(dplyr);
library(hash);
library(xlsx);
pt52_A = read.xlsx(file = "   ", sheetName = "Sheet1");
pt52_A = pt52_A[,c(1,6,7,16,21)];
pt52_A = filter(pt52_A, log2FoldChange != "-Inf");
pt52_B = read.xlsx(file = "   ", sheetName = "Sheet1");
pt52_B = pt52_B[,c(1,6,7,16,21)];
pt52_B = filter(pt52_B, log2FoldChange != "-Inf");
pt55_3 = read.xlsx(file = "   ", sheetName = "Sheet1");
pt55_3 = pt55_3[,c(1,6,7,16,21)];
pt55_3 = filter(pt55_3, log2FoldChange != "-Inf");
pt55_7 = read.xlsx(file = "   ", sheetName = "Sheet1");
pt55_7 = pt55_7[,c(1,6,7,16,21)];
pt55_7 = filter(pt55_7, log2FoldChange != "-Inf");
setwd(workingdir1);
Entrezid_list = list.files();
samplecor = hash(keys = Entrezid_list, values = c("pt52_A", "pt52_B", "pt52_B", "pt55_7","   ", "   ",
"   "));
```

```
for(i in 1:length(Entrezid_list)){
 setwd(workingdir1);
 entrez = read.csv(Entrezid_list[i]);
 entrez = entrez[,c(3,4)];
 a = samplecor[[Entrezid_list[i]]];
 entrez$Entrez_geneID = as.character(entrez$Entrez_geneID);
 if(a == "pt52_A")
  merged = merge(entrez, pt52_A, by = "Entrez_geneID");
 if(a == "pt52_B")
  merged = merge(entrez, pt52_B, by = "Entrez_geneID");
 if(a == "pt55_3")
  merged = merge(entrez, pt55_3, by = "Entrez_geneID");
 if(a == "pt55_7")
  merged = merge(entrez, pt55_7, by = "Entrez_geneID");
 merged = subset(merged, merged$pval < 0.05);
 if(dim(merged)[1] == as.integer(0)) next;
 setwd(workingdir2);
 write.csv(merged,
           file = paste(substring(Entrezid_list[i],
                                  1, nchar(Entrezid_list[i])-4),
                                  "_filtered.csv", sep = ""));
 print("done");
}
```

3.1.4 Identify Pathway

```
library(KEGGREST);
library(dplyr);
library(pathview);
for(q in 1:length(HubGene_List)){
 setwd(workingdir0);
 test = read.csv(file = HubGene_List[q]);
 test = test[,-1];
```

```
  test = filter(test, KEGG != "-");
  if(dim(test)[1] == as.integer(0)){
    next;
  }
  test = cbind(test, KEGG_id = 0);
  test = cbind(test, pathway_id = 0);
  for(i in length(test$Entrez_geneID)){
    test$Entrez_geneID = paste("ncbi-geneid", test$Entrez_geneID, sep = ":");
  }
  for(i in 1:length(test$Entrez_geneID)){
    test$KEGG_id[i] = keggConv("pti", test$Entrez_geneID[i]);
  }

  for(i in 1:length(test$Entrez_geneID)){
    pathway = keggLink("pathway", test$KEGG_id[i]);
    if(length(pathway) == 0){
      test$pathway_id[i] = NA;
    }else{
    pathwayid = ""
    for(j in 1:length(pathway)){
      pathwayid = paste(pathwayid, pathway[j], sep = "/");
    }
    test$pathway_id[i] = substring(pathwayid, 2, nchar(pathwayid));
    }
  }
  setwd(workingdir2);
  write.csv(test, file = paste(substring(HubGene_List[q], 1,
                                          nchar(HubGene_List[q])-4),
                                          "_pathway.csv", sep = ""));
}
```

3.2 R codes of plot

Fig.8 Heat map depiction of the topological overlapping matrix (TOM).

```
load(file = "TOMplot.Rdata");
View(plotTOM)
plotTOM = 1-plotTOM;
sizeGrWindow(9,9)
pdf(file = "TOMplot.pdf")
TOMplot(plotTOM, geneTree, moduleColors, main = "Network heatmap plot, all genes")
```

Other heat maps (From Figure9 to Figure 10) use the data generated in WGCNA analysis to draw heat maps.

Fig.11 Gene significance vs. connectivity scatter plot.

```
GSPvalue = as.data.frame(corPvalueStudent(as.matrix(geneTraitSignificance), nSamples));
names(geneTraitSignificance) = paste("GS.", names(tempdf), sep = "");
names(GSPvalue) = paste("p.GS", names(tempdf), sep = "");
colorlevels = unique(moduleColors);
pdf(paste("GSvsDegree_", names(tempdf), ".pdf", sep = ""), width = 14, height = 8);
par(mfrow = c(3,4));
par(mar = c(5,5,3,3));
for(i in c(1:length(colorlevels))){
whichmodule = colorlevels[[i]];
restrict1 = (moduleColors == whichmodule);
verboseScatterplot(alldegrees$kWithin[restrict1],
geneTraitSignificance[restrict1,1],
col = moduleColors[restrict1],
main = whichmodule,
xlab = "Connectivity", ylab = "Gene Significance",
abline = T);
}
dev.off();
print("done");
}
```
